# Effects of Boiling Drinking Water on Diarrhea and Pathogen-Specific Infections in Low- and Middle-Income Countries: A Systematic Review and Meta-Analysis

**DOI:** 10.4269/ajtmh.17-0190

**Published:** 2017-09-05

**Authors:** Alasdair Cohen, John M. Colford

**Affiliations:** 1 Division of Epidemiology, School of Public Health, University of California at Berkeley, Berkeley, California;; 2 Department of Environmental Science, Policy and Management, University of California at Berkeley, Berkeley, California

## Abstract

Globally, approximately 2 billion people lack microbiologically safe drinking water. Boiling is the most prevalent household water treatment method, yet evidence of its health impact is limited. To conduct this systematic review, we searched four online databases with no limitations on language or publication date. Studies were eligible if health outcomes were measured for participants who reported consuming boiled and untreated water. We used reported and calculated odds ratios (ORs) and random-effects meta-analysis to estimate pathogen-specific and pooled effects by organism group and nonspecific diarrhea. Heterogeneity and publication bias were assessed using *I*^2^, meta-regression, and funnel plots; study quality was also assessed. Of the 1,998 records identified, 27 met inclusion criteria and reported extractable data. We found evidence of a significant protective effect of boiling for *Vibrio cholerae* infections (OR = 0.31, 95% confidence interval [CI] = 0.13–0.79, *N* = 4 studies), *Blastocystis* (OR = 0.35, 95% CI = 0.17–0.69, *N* = 3), protozoal infections overall (pooled OR = 0.61, 95% CI = 0.43–0.86, *N* = 11), viral infections overall (pooled OR = 0.83, 95% CI = 0.7–0.98, *N* = 4), and nonspecific diarrheal outcomes (OR = 0.58, 95% CI = 0.45–0.77, *N* = 7). We found no evidence of a protective effect for helminthic infections. Although our study was limited by the use of self-reported boiling and non-experimental designs, the evidence suggests that boiling provides measureable health benefits for pathogens whose transmission routes are primarily water based. Consequently, we believe a randomized controlled trial of boiling adherence and health outcomes is needed.

## INTRODUCTION

Across low- and middle-income countries (LMICs), close to 2 billion people lack reliable access to microbiologically safe drinking water, and approximately 500,000 people, mostly children, die annually due to unsafe or insufficient drinking water.^1–6^ In the most recent (2015) Global Burden of Disease study,^7^ unsafe water was ranked 14th among global health risks. Point-of-use household water treatment (HWT) technologies are often recommended when reliable access to safe water is limited. Filtration (ceramic, biosand, and micro), chlorination (with/without flocculation), solar disinfection, and ultraviolet (UV) disinfection are the primary HWT technologies currently promoted in LMICs. When used correctly, these HWT technologies effectively improve drinking water quality and can reduce related morbidity and mortality.^8–10^ However, after decades of extensive promotion efforts, achieving the widespread and sustained adoption of these HWT technologies remains a challenge.^11–15^

Boiling is the most commonly used reported HWT method globally, with an estimated 1.2 billion users (∼70% of all HWT users).^14,16,17^ The reported use of boiling is particularly widespread in many Asian nations, including China, where as many as 85% of rural residents report boiling drinking water,^16^ as well as an estimated 95% in Mongolia and 91% in Indonesia and Vietnam.^14^ Compared with HWT products such as chlorine or filters, however, relatively few health or water, sanitation, and hygiene (WASH) studies have focused on boiling specifically. Among the boiling-focused studies, most evaluated boiling and water quality outcomes, but not health outcomes. Water-quality-focused studies in Cambodia, Guatemala, India, Indonesia, Peru, and Vietnam all found significant post-boiling reductions of fecal contamination indicators.^18–23^ Although boiling is straightforward to use and microbiologically effective, as with other HWT methods, its effectiveness depends on correct and consistent use. Boiled water is also susceptible to recontamination, and the fuels used to boil water in LMIC settings often produce household air pollution (HAP).^23–26^ In addition, there is a potential for injury via skin exposure to hot or boiling water.

The relative paucity of boiling-focused health research has not gone unnoticed. For example, a comprehensive review of point-of-use water treatment technologies and methods for use in emergencies cited a “lack of epidemiological confirmed health impact” for boiling,^27^ and a recent World Health Organization report noted that there is relatively little research on boiling’s effectiveness for diarrheal reduction.^5^ Moreover, as noted in the most recent Cochrane Review on interventions to improve drinking water quality, no randomized controlled trials (RCTs) have been conducted to evaluate boiling.^28^

Similarly, although there are a number of systematic reviews and summary articles on the use of chlorination, filtration, and solar disinfection,^3,12,13,29–31^ as far as we are aware, there are no such reviews focused on boiling and health outcomes, or on boiling and water quality, specifically (in part because some previous reviews only considered experimental study designs as eligible). Furthermore, these reviews, and most of the WASH studies they are based on, tend to use diarrheal disease as the primary health outcome. Because many pathogens result in diarrheal symptoms, these analyses do little to clarify the relative effectiveness of different HWT methods for exposure to specific pathogens or organism groups.

A clearer understanding of boiling’s impact on water-related disease prevention is needed. We conducted this systematic review and meta-analysis to bring together the evidence on boiling and health outcomes in LMICs. This study is also one of the few such reviews to attempt to estimate pooled effects for specific pathogens and organism groups,^32,33^ as well as for nonspecific diarrheal disease outcomes.

## MATERIALS AND METHODS

### Search strategy and selection criteria.

To identify potentially eligible studies, we searched four online databases: PubMed/MEDLINE, EMBASE, Web of Science, and the Cochrane Library. Search terms were selected with the goal of finding all articles that might potentially address health outcomes associated with the boiling of drinking water in LMICs. Four sets of search terms were used to identify all articles focused on drinking water, drinking water treatment (including, but not limited to, boiling), health outcomes known to be associated with the consumption of contaminated drinking water, and the names and alternate names/spellings of all LMICs. Because some search engines retrieve fewer results when truncation is used,^34^ we included all possible word variants in our lists of search terms (e.g., rather than using “boil*,” we searched for “boils,” “boiled,” and “boiling”). The search terms, sets, and an explanation of the Boolean operators used are provided in Supplemental Table 1.

The final database literature searches were conducted on January 21, 2016 (the complete searches used for each database are provided in Supplemental Tables 2–5). No restrictions were put in place with regard to publication date, type, or language. In addition, a hand-search was conducted by consulting the reference sections of articles already known to discuss boiling and drinking water treatment as well as a targeted search for papers using Google Scholar (grey literature was not included). Following the convention to define eligibility with reference to the population/s, study/intervention, comparisons, and outcomes of interest,^35^ studies were considered eligible if they included human participants in LMICs; measured infectious health outcomes (disease occurrence) due to pathogens with at least one water-related transmission route; and there was a comparison, or data which could be used to make a comparison, for such outcomes between participants reporting to drink boiled water and those reporting to drink non-boiled/untreated water (any study design with data for such a comparison). We did not include unpublished studies.

After the databases were searched, the results were exported and compiled using the reference management software Endnote (version X7; Thomson Reuters, New York, NY). Duplicates were removed using Endnote’s automated process, followed by a manual search to identify and remove additional duplicates. For the initial record screening step, to avoid inadvertent bias from viewing author name/s, publication type, journal names, and so on, only the record titles and abstracts were reviewed. Titles/abstracts that did not mention boiling but did describe studies focused on drinking water treatment and health outcomes were retained in the hopes that subgroup or control group data related to boiling and health outcomes were reported in the full text. One reviewer (Alasdair Cohen) screened all the titles and abstracts (when available) to determine which were eligible for full-text review. Titles and abstracts from a randomly selected sample of 5% of the initial records were screened by a second reviewer (John M. Colford) and inter-rater reliability was assessed. Similarly, after full-text review (by Alasdair Cohen), 15% of the full-text articles were randomly selected and reviewed for eligibility (by John M. Colford).

### Data extraction, calculation, and derivation protocols.

For each eligible study with extractable data associated with the health effects of consuming boiled drinking water, the following summary information was extracted from the full text if available: country where the study was conducted, province/state/region within the country, study population (rural, urban, mixed, etc.), study type and design, year/s the study was conducted, study duration in months, total number of individuals (and/or households) sampled, age/s of participants, whether a random sampling/selection process was used, whether the sampling/method was described, the health outcome/s assessed, whether a protocol for outcome assessment was described, and whether the outcome assessment was direct or based on self-report.

To extract or calculate odds ratios (ORs), such that values < 1.0 would signify a reduction in disease associated with the consumption of boiled drinking water, as well as lower and upper 95% confidence intervals (95% CI) from each study for our meta-analysis, our guiding principle was to use the best available data in all cases. When the data were provided, or could be calculated, we constructed 2 × 2 tables and calculated ORs and 95% CIs. If these values aligned with those reported in the text, we used our calculations. For studies that reported the OR but did not provide sufficient data to construct a 2 × 2 table, we used their reported estimates. When the reported OR reference group was those who did not boil their water, we used the reported upper and lower 95% CI to back-calculate the standard error (SE) of the log(OR) to derive 95% CIs for those who boiled (using the inverse of the reported OR). Similarly, in cases where the authors rounded the 95% CI to one decimal place and the data were available, we back-calculated the SE to derive more precise 95% CIs.

When authors provided adjusted estimates, we recorded them in our dataset and also calculated unadjusted estimates when the data were available, but only used the reported adjusted estimates for the primary analyses presented here. For matched case–control studies, we always used the reported matched odds ratio (MOR) when provided, back-calculating to derive the MOR and 95% CI for the boiling group if needed. If the authors only reported a risk ratio (RR), we treated it as an OR. For additional details, see the Supplemental Dataset 1 (“comments” in the data cells provide the table and/or page number/s where we found the data from each study).

For our analyses of possible publication bias, for those studies where we had to transform and back-calculate 95% CIs and the resulting SEs of the upper and lower 95% CI were not equal, we used the arithmetic mean of the upper and lower values to estimate the boiling SE of the log(OR) (these instances are marked with yellow font in column “AE” of Supplemental Dataset 1). Following data extraction of all eligible studies (by Alasdair Cohen), 30% were randomly selected for data extraction/derivation by a second reviewer (John M. Colford). All extracted data and related calculations were reviewed and discussed by both reviewers.

### Data analysis.

We used meta-analysis to estimate pooled effects of boiling drinking water on health outcomes. Because of the differences in pathogenesis for the various disease outcomes assessed in the studies, we chose not to estimate an overall pooled effect for boiling across all disease outcomes. Rather, we created outcome groups by combining studies that assessed bacterial, helminthic, protozoal, and viral infections, as well as diarrheal outcomes with no specified etiology. Because some authors adjusted for covariates and others did not, we used the most adjusted estimates when available. Using only unadjusted outcome effects tended to result in more protective pooled estimates, thus our use of the adjusted estimates when available resulted in more conservative point and pooled estimates overall (unadjusted estimates are provided in Supplemental Dataset 1).

Given our expectation of inter-study variability (due to differences in study design, data collection methods, testing protocols, etc.) and random error, we used meta-analysis with random-effects-based weighting. Because of the known power issues with regard to detecting heterogeneity in meta-analyses generally, and when using subgroups specifically, in addition to using Mantel–Haenszel estimates of heterogeneity, we used the *I*^2^ statistic to assess the degree of variation in subgroups which could be attributed to inter-study heterogeneity.^36^ For studies where the authors provided adjusted effect estimates, we performed meta-analyses using only the adjusted effect estimates.

To further examine heterogeneity and identify potential confounders, we used meta-regression analysis with random effects (controlling for the variance within and between studies) to examine the impact of various study characteristics on the log(OR) for boiling. Specifically, we regressed the log(OR) for boiling on the total number of participants (or households), participant age, whether the study participants lived in rural areas or not, whether the study was an outbreak investigation or not, study duration, whether any type of random selection or sampling method was used to select participants, and lastly, whether the primary health outcome was assessed via self-report or measured directly, meaning infection was confirmed via analysis of stool and/or serum samples (e.g., with enzyme-linked immunosorbent assay, microscopy, direct smear, cell culture, polymerase chain reaction). Because of the relatively small number of studies available for many organism groups, we also estimated adjusted *P* values using a Monte Carlo permutation test (with 1,000 random permutations). To attempt to evaluate study quality/bias, we scored each study on a variety of criteria and then aggregated the resulting six components into a composite index which we converted to a 10-point scale to assign grades to each study (we adapted the criteria and grading approach from two recent reviews^2,37^; see Supplemental Table 6). We then incorporated these quality classifications into an additional meta-regression analysis. Because one might expect baseline exposure and boiling adherence to be higher during outbreak events, pooled estimates that included outbreak investigation studies were estimated with and without outbreak data.

Funnel plots were created to visually assess the extent of potential publication bias in combination with the use of Egger’s test.^38^ Though regressing log(OR)s on corresponding SEs may be prone to false positives, we used Egger’s test (at a 95% CI) to attempt to quantitatively assess the degree of potential publication bias (because we did not have complete 2 × 2 data for all studies, we were limited with regard to the use of other such tests). We analyzed each organism group in isolation and conducted an exploratory analysis stratifying by study design.

All analyses were conducted using STATA (v13.1; StataCorp, College Station, TX). A completed PRISMA^39^ checklist is provided in Supplemental Table 7.

## RESULTS

**Figure 1.**
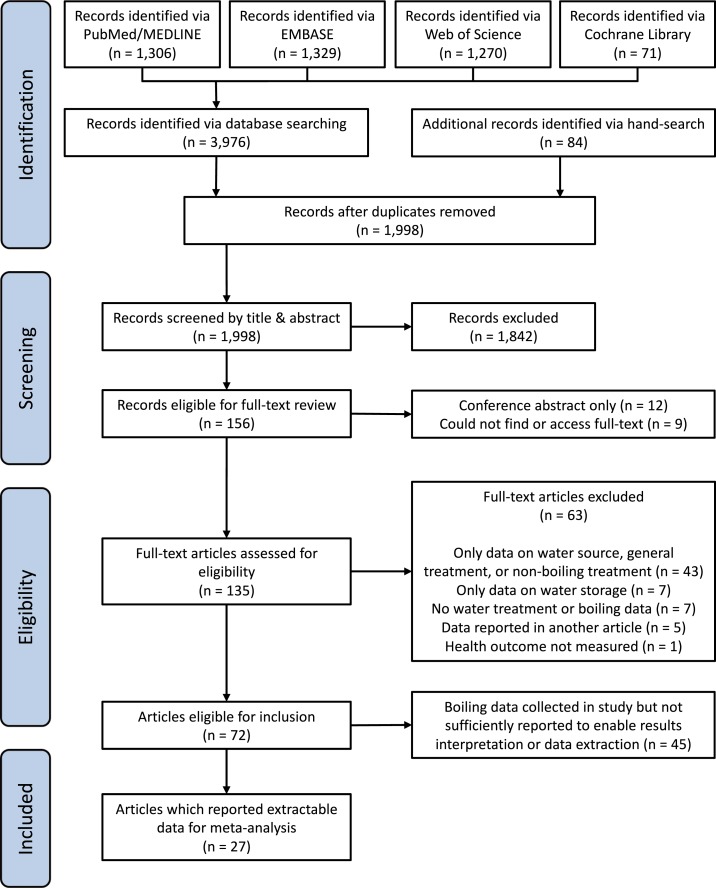
Flowchart of the systematic review process used to identify eligible studies. This figure appears in color at www.ajtmh.org.

After removing duplicates across the four databases and hand-search results, 1,998 records were identified (see Figure 1). Screening by titles and abstracts resulted in the selection of 156 records for full-text review. For the randomly selected subset of 5% (*N* = 100) records, there was 93% agreement between the two reviewers (kappa = 0.55), which was considered sufficient given the broad inclusion criteria used for the initial screening. One hundred thirty-five full-text articles were found, published during 1955–2015, with 91% (*N* = 123) in English, 6% (*N* = 8) in Spanish, and 3% (*N* = 4) in Chinese (both reviewers read English and Spanish, and Alasdair Cohen’s Chinese reading ability was sufficient for this review). After full-text review (by Alasdair Cohen), 63 articles were deemed ineligible.^40–102^ For the randomly selected subset of 15% (*N* = 23) full-text articles reviewed (by John M. Colford), there was 100% agreement with regard to eligibility (none of these randomly selected articles were published in Chinese). Of the 72 articles eligible for inclusion, 27 reported extractable boiling and health outcome data,^103–129^ whereas 45 did not report sufficient data for interpretation or extraction.^130–174^ To check the accuracy of data extraction (by Alasdair Cohen), 30% (*N* = 8) of these articles were randomly selected and the second reviewer (John M. Colford) performed independent data extraction; this resulted in 100% agreement.

As discussed earlier, the guiding protocol was to use the best available data, and so when presented with a decision we always used the more conservative and/or broadly relevant data. In the interests of consistency and replicability, we only used data provided in the papers, rather than using effect estimates reported elsewhere or non-published data to which we had access (or by contacting authors directly). For example, in Núñez and others,^121^ we used the verified “Hierve el agua (verificado),” rather than unverified boiling data. Similarly, for our published research on boiling in China,^175^ since we did not publish the diarrheal RR for all boiling methods, we used the published RR for boiling with metal pots, rather than electric kettle-based boiling (since pot-based boiling is more broadly relevant). In addition, because we could not assume that the water was heated to boiling for all reported boiling cases in all studies, and because pathogen inactivation can occur at temperatures as low as ∼55–60°C, depending on the altitude, pathogens, and boiling durations,^176^ we considered Iijima and others’^115^ study on water pasteurization eligible.

The 27 articles from which data were extracted were published over the years 1992–2015, with 81.5% (*N* = 22) published in English and 18.5% (*N* = 5) in Spanish. Studies were conducted in countries around the world, with multiples studies in India (*N* = 4), Malaysia (*N* = 4), Cuba (*N* = 3), Peru (*N* = 3), and China (*N* = 2). Slightly more than half of the articles (55.6%, *N* = 15) described results from cross-sectional designs. Of the studies, 40% (*N* = 11) were conducted with participants from rural areas, 22% (*N* = 6) urban, and 37% (*N* = 10) mixed rural and urban. The median number of participants was 283, with a mean of 1,500 (SD = 2,836, *N* = 25) and the median duration of the study or data collection was 4 months, with a mean of 11.1 months (SD = 18.8, *N* = 27). Health outcomes were measured directly in 74% of the articles (*N* = 20), measured and reported in 11% (*N* = 3), and only reported in 15% (*N* = 4) (the specific methods used for direct measurement in each study are provided in Supplemental Dataset 1). See Table 1 for a summary of the study characteristics, specific outcomes, and the data sources and methods used to derive effect estimates.

**Table 1:** Characteristics of studies included in meta-analysis, organized by organism group

	Specific pathogen or outcome	First author	Published year	Country where study conducted	Year/s study conducted	Study duration (months)	Rural or urban	Number of participants (number of households)	Participant age	Study design	Random selection or sampling used	Outcome measured or reported	OR data source
Bacteria	*Helicobacter pylori*	Lee	2012	Malaysia	2002–2008	72	R&U	161	A	CC	U	M	OR, C
	*Salmonella typhi*	Sharma	2009	India	2005–2006	17	R&U	246	M	MCC	Y	M	MOR, R
	*Vibrio cholerae*	Cardenas	1993	Colombia	1991–1992	10	R&U	(209)	M	CS	Y	*R*	OR, R
	*V. cholerae*	Fredrick	2015	India	2012	1	R&U	154	M	MCC	U	*M&R*	MOR, RT
	*V. cholerae*	Ries	1992	Peru	1991	1	U	150	M	MCC	U	*M*	MOR, R
	*V. cholerae*	Weber	1994	Ecuador	1991	1	U	189	C	CC	U	*M*	OR, C
Helminths	*Ascaris*	Gunawardena	2004	Sri Lanka	2000	6	R	176	M	CS	Y	M	OR, RAT
	*Strongyloides* sterocoralis	Herrera	2006	Peru	2003	2	R	100	M	CC	U	M	OR, C
	Ascaris, *Trichuris*, hookworm, and multiple	Wordemann	2006	Cuba	2003 & 2004	2	R&U	1320	C	CS	Y	M	OR, RT
	Multiple	Al-Delaimy	2014	Malaysia	2012	4	R	498	C	CS	N	M	OR, C
Protozoa	*Blastocystis*	Carrero	2013	Columbia	–	1	R&U	50	C	CS	N	M	OR, C
	*Blastocystis*	Li	2007	China	–	1	R	283	M	CS	Y	M	OR, RT, & RAT
	*Blastocystis*	Rondon	2003	Peru	1999	3	R&U	144	M	CC	U	M	OR, C
	*Cryptosporidium parvum*	Sarkar	2014a	India	2008–2013	60	U	580	C	NCC	U	M	OR, C, & RA
	*Giardia*	Bello	2011	Cuba	2003	6	R&U	351	C	CC	N	M	OR, C, & RAT
	*Giardia*	Choy	2014	Malaysia	2011–2013	22	R	1330	M	CS	Y	M	OR, C, & RAT
	*Giardia*	Nunez	2003	Cuba	–	18	U	119	C	L	U	M	OR, C
	*Entamoeba histolytica*, *Giardia*, and multiple	Wordemann	2006	Cuba	2003 and 2004	2	R and U	1320	C	CS	Y	M	OR, RT
	Multiple	Marcano	2013	Venezuela	2012	2	U	324	M	CS	U	M	OR, C
Viruses	Hepatitis E	Aggarwal	2002	India	1998	5	R and U	1088	M	CS	Y	*M*	RR, R
	Hepatitis E	Corwin	1995	Indonesia	1993	1	R	445	M	CS	U	M	OR, C
	Rotavirus	Sarkar	2014b	Bangladesh	1993–1997	48	U	9879	C	CCh	U	M	OR, C, and RA
	Rotavirus	Sarkar	2014b	Bangladesh	2008–2012	48	U	6204	C	CCh	U	M	OR, C, and RA
Diarrhea	Nonspecific diarrhea	Cardenas	1993	Colombia	1991–1992	10	R and U	(209)	M	CS	Y	*R*	OR, R
	Nonspecific diarrhea	Cifuentes	1998	Mexico	1992	5	R	9435	M	CS	U	M	OR, C
	Nonspecific diarrhea	Cohen	2015	China	2013	1	R	(450)	M	CS	Y	R	RR, R
	Nonspecific diarrhea	Iijima	2001	Kenya	1995	4	R	3420	M	CS	U	R	OR, C
	Nonspecific diarrhea	Kelly	1997	Zambia	1995–1996	5	R and U	6702	A	CS	U	M and R	OR, R
	Nonspecific diarrhea	Knight	1992	Malaysia	1989	2	R	196	C	MCC	Y	M and R	OR, RAT
	Nonspecific diarrhea	Psutka	2013	Kiribati	2011	1	R	153	C	CS	Y	R	RR, RT

Rural or urban: R = rural, U = urban; participant age: C = children (age < 18), A = adults (age > 18), M = mixed (all ages), study design: CS = cross-sectional, CC = case–control, MCC = matched case–control, NCC = nested case–control, L = longitudinal, CCh = case-cohort; random selection: Y = yes, N = no, U = unclear; outcome measurement: M = measured directly (details in Supplemental Dataset 1, column CG), R = based on self-report. Outbreak investigations marked in italics (*N* = 6); OR data source: RR = risk ratio, OR = odds ratio, MOR = matched odds ratio, R = reported, T = transformed, A = adjusted, C = calculated (2 × 2 data).

**Figure 2.**
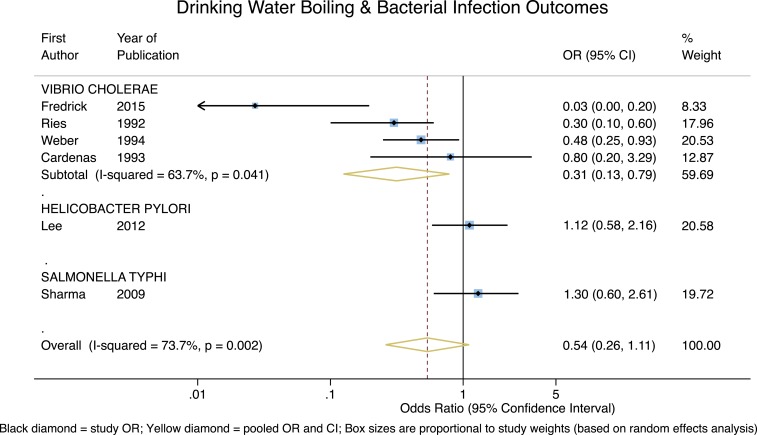
Forest plot for studies measuring bacterial outcomes. This figure appears in color at www.ajtmh.org.

Disease outcomes were organized into bacterial, helminthic, protozoal, and viral groups, as well as nonspecific diarrheal disease outcomes. For bacterial outcomes, as shown in Figure 2, boiling drinking water is associated with a significant and highly protective effect for *Vibrio cholerae* (OR = 0.31, 95% CI = 0.13–0.79, *P* = 0.01), though the heterogeneity is somewhat high (*I*^2^ = 63.7%). However, effects from the single studies of *Helicobacter pylori* and *Salmonella typhi* are neither protective nor significant (*P* = 0.74 and *P* = 0.49, respectively). Consequently, although the pooled estimate for these bacterial outcomes is protective, it is not significant (overall OR = 0.54, 95% CI = 0.26–1.11, *P* = 0.09) and the heterogeneity was high (*I*^2^ = 73.7%). In addition, all four *V. cholera* studies were outbreak investigations; with those studies removed, the pooled estimate for the remaining two bacterial outcomes is neither protective nor significant (overall OR = 1.19, 95% CI = 0.73–1.95, *P* = 0.48), with essentially zero heterogeneity.

**Figure 3.**
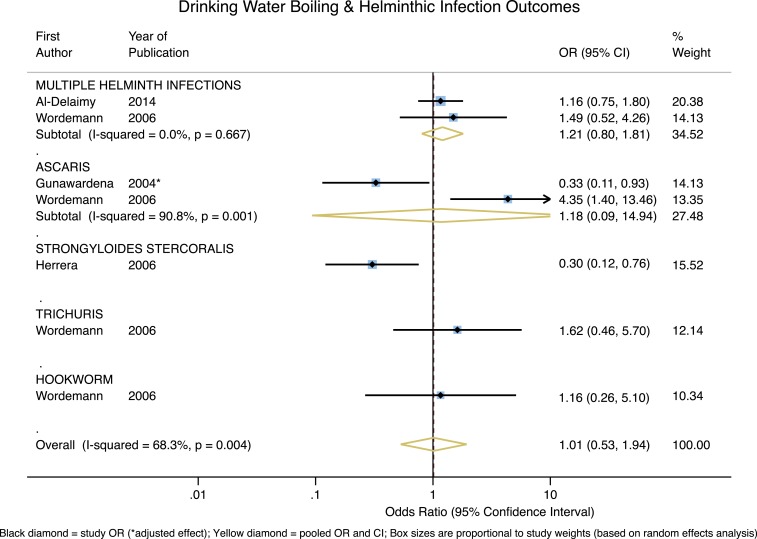
Forest plot of studies measuring helminthic outcomes. This figure appears in color at www.ajtmh.org.

As shown in Figure 3, across helminth infection outcomes, the only significant protective effect associated with boiling is for the single study reporting on *Strongyloides stercoralis* (OR = 0.30, 95% CI = 0.12–0.76, *P* = 0.01). The two studies of *Ascaris* reported significant effects on either side of the null, and across helminthic outcomes the pooled effect estimate is essentially null (overall OR = 1.01, 95% CI = 0.53–1.94, *P* = 0.97) with high heterogeneity (*I*^2^ = 68.3%).

**Figure 4.**
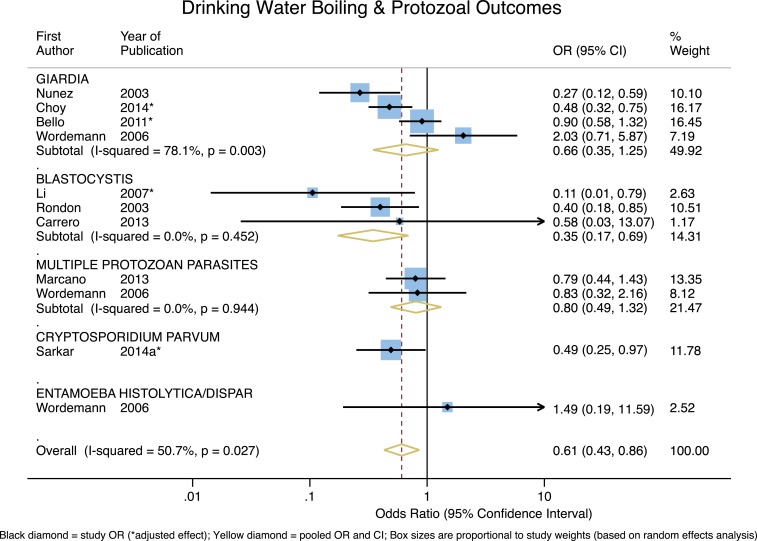
Forest plot of studies measuring protozoal outcomes. This figure appears in color at www.ajtmh.org.

For studies that measured protozoal infections, the pooled effect across the four studies of *Giardia* suggests that boiling may have a protective effect, but it is not significant (OR = 0.66, 95% CI = 0.35–1.25, *P* = 0.20) and the heterogeneity is quite high (*I*^2^ = 78.1%). Based on the three available studies, boiling is associated with a significant and strong protective effect for *Blastocystis* (OR = 0.35, 95% CI = 0.17–0.69, *P* = 0.003), and the variation in the effects does not appear to be attributable to heterogeneity; the heterogeneity statistic also suggests that the underlying effect is relatively constant (*P* = 0.45). For the two studies that measured the effect of boiling on infection with multiple protozoan parasites, the pooled effect is protective, but not significant (OR = 0.80, 95% CI = 0.49–1.32, *P* = 0.39) and there is no significant heterogeneity. The one study on *Cryptosporidium parvum* found a strong and significant protective effect of boiling. The single study on *Entamoeba histolytica* did not report a protective effect. The overall pooled effect of boiling on protozoan infections was protective and significant (overall OR = 0.61, 95% CI = 0.43–0.86, *P* = 0.005) with moderate heterogeneity (*I*^2^ = 50.7%) (see Figure 4).

**Figure 5.**
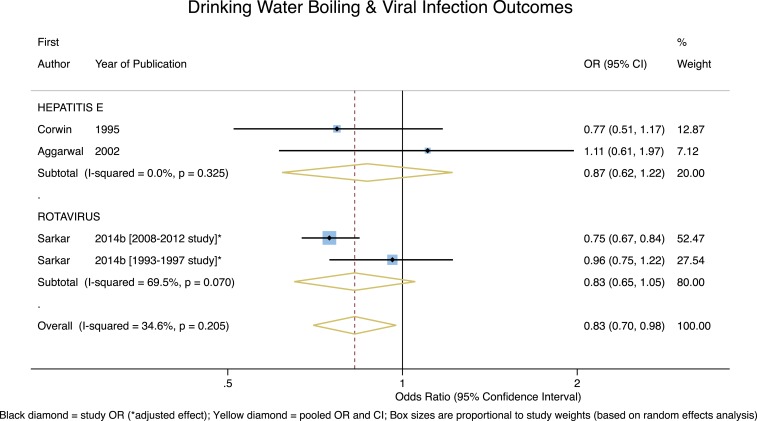
Forest plot of studies measuring viral outcomes. This figure appears in color at www.ajtmh.org.

For viral outcomes, as can be seen in Figure 5, though both of the pooled effect estimates for the two studies of *Hepatitis E* and the two studies of *Rotavirus* suggested boiling may be protective, neither were significant (*P* = 0.42 and *P* = 0.12, respectively). Although the overall pooled estimate for all viral infection outcomes was both protective and significant (overall OR = 0.83, 95% CI = 0.70–0.98, *P* = 0.02), with low-to-moderate heterogeneity (*I*^2^ = 34.6%), this result was due to the large weighting (52.5%) from the Sarkar 2008–2012 study. With the one outbreak investigation (Aggarwal) excluded, the overall pooled estimate for viral infection outcomes remains protective and significant (overall OR = 0.81, 95% CI = 0.68–0.95, *P* = 0.01), with low-to-moderate heterogeneity (*I*^2^ = 39.1%).

**Figure 6.**
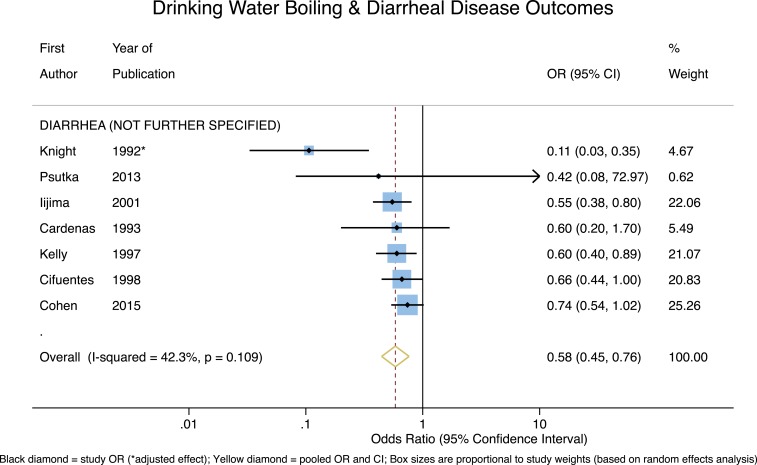
Forest plot of studies measuring non-specific diarrheal outcomes. This figure appears in color at www.ajtmh.org.

Finally, for the studies with nonspecific diarrheal disease outcomes, shown in Figure 6, the pooled effect estimate indicates that reported boiling of drinking water is significant and strongly protective (OR = 0.58, 95% CI = 0.45–0.77, *P* < 0.001), and with only moderate heterogeneity (*I*^2^ = 42.3%). With the outbreak investigation (Cardenas) removed, the pooled effect estimate remains significant and strongly protective (OR = 0.58, 95% CI = 0.43–0.78, *P* < 0.001), but with slightly higher heterogeneity (*I*^2^ = 51.9%).

Results of the meta-regression analyses for studies with protozoal and diarrheal outcomes indicated that none of the tested variables significantly impacted the effect estimates for boiling (and except for the covariate for total participants in the protozoal outcomes model, none of the Monte Carlo permutation derived *P* values fell below the 0.05 threshold). Because of the relatively small number of studies in each organism group, there was an insufficient number of observations and/or too much collinearity to estimate covariate coefficients for studies with bacterial, helminthic, and viral outcomes. See Supplemental Tables 8 and 9 for model results.

With regard to possible publication bias, Funnel plots for each outcome group were visually inspected and, aside from nonspecific diarrheal outcomes, none indicated likely publication bias (see Supplemental Figures 1–5). Similarly, Egger’s test did not indicate evidence of a “small study” effect for bacterial outcomes (*P* = 0.17), nonspecific diarrheal outcomes (*P* = 0.18), helminthic outcomes (*P* = 0.96), protozoal outcomes (*P* = 0.78), or viral outcomes (*P* = 0.31). In an exploratory effort, we also examined a Funnel plot of all study outcomes (Supplemental Figure 6) which likewise did not indicate publication bias (Egger’s test *P* = 0.26). After stratifying by study design (Supplemental Figures 7 and 8), there did not appear to be publication bias for the cross-sectional outcomes, though there were indications of publication bias for the other study designs (which were mostly case–controls; Egger’s test *P* = 0.30 and *P* = 0.03, respectively).

Concerning estimated study quality/bias, four studies (11%) received a low grade, 10 (29%) a medium grade, and 21 (60%) a high grade (see Supplemental Table 10). For none of the pathogen-specific outcomes were there more than two studies with significant pooled ORs which also fell into different quality/bias classifications (see Supplemental Table 11). For diarrheal outcomes, the pooled ORs for the studies assessed to be of low and medium quality were protective and significant, but approximately equal (though the CI was tighter for the medium-quality studies: low-quality studies OR = 0.60, 95% CI = 0.40–0.89, *N* = 2; medium-quality studies OR = 0.60, 95% CI = 0.50–0.78, *N* = 3); the pooled OR for the high-quality diarrheal studies was the lowest, but not significant (high-quality studies OR = 0.31, 95% CI = 0.05–2.03, *N* = 2).

## DISCUSSION

The results of our systematic review and meta-analyses suggest that boiling’s protective effect is stronger for some pathogens and organism groups than for others. These findings appear to align with current understandings of transmission pathways for different pathogens and the role of drinking water treatment,^177^ such that for those pathogens with primarily water-related transmission routes, reported boiling appears to be protective.

One potential complication with regard to understanding boiling’s differential effect on specific pathogens is related to whether water is actually boiled, or merely heated.^176^ Although boiling water at 100°C (at sea level) should inactivate all known pathogenic organisms in the water, at temperatures less than 100°C rates of pathogen inactivation vary by temperature, duration, and the organism in question (as altitude increases the boiling point decreases).^176,178^ For example, at sea level, a one log reduction in the concentration of *S. typhi* can be achieved in ∼77 seconds at 55°C, or approximately 4 seconds at 60°C, whereas for pathogenic *Escherichia coli* (O157:H7) a one log reduction is achieved in ∼223 seconds at 55°C, or ∼67 seconds at 60°C.^179^ Inactivation levels for a protozoa, such as *C. parvum*, also vary considerably based on the temperature and exposure duration.^180^

When boiling is promoted, generally or in the context of boiling advisories, the usual recommendation is to bring water to a rolling boil since this treatment endpoint can be easily observed.^178^ If we assume that most study participants who reported boiling did bring their water to a rolling boil, then—putting aside for the moment issues of safe storage, secondary contamination, and consistent adherence—full pathogen inactivation is to be expected.^176^ In this respect, boiling is superior to other HWT methods wherein the susceptibility of pathogens in drinking water varies based on the method of treatment, water turbidity, and the pathogen in question.^177^ There is also considerable variation in inactivation effects for different pathogens depending on which specific variant of given HWT is used (e.g., the variable effectiveness of different forms of chlorine on *E. coli*).^181^

Looking to our results for bacterial outcomes, *V. cholerae* bacteria are transmitted via the fecal–oral route with contaminated drinking water serving as the most common transmission pathway^182^; it is, therefore, not surprising that boiling appears to provide such a strong preventative effect. For *H. pylori*, on the other hand, the global prevalence is relatively high and quite varied geographically, infection is often asymptomatic, and though transmission remains poorly understood, the oral–oral route is suspected to be the primary method of transmission^183,184^; as such, the lack of evidence for boiling’s preventative effect is perhaps not surprising. *Salmonella typhi*, on the other hand, is also spread via the fecal–oral route, and foodborne transmission appears to be more common than water-related transmission,^185^ hence boiling alone would not be expected to reliably prevent infection.

This same logic may be applied to pathogens in the helminthic, protozoal, and viral outcome groups. Broadly speaking, helminth infections are usually the result of consuming foods contaminated with feces or soils that contain helminth eggs, or via contact with exposed skin.^58,177^ That water is not the primary transmission route for helminthic infections is consistent with our overall null findings for the impact of boiling on various helminthic pathogens (aside from the significant protective effect associated with *S. stercoralis*, based on one study). Though water is not the only transmission route for protozoal infections, reported boiling appears to be broadly protective across specific protozoa. Boiling’s highly protective effect for *C. parvum*, though based only on one study, is noteworthy given *C. parvum’s* extreme resistance to chlorine inactivation.^186^ The apparent effectiveness of boiling on viral outcomes may also be of interest, given that enteric viruses are primarily transmitted through the fecal–oral route via contaminated food or water, though airborne transmission has also been reported.^187,188^ The possible protective effect of boiling for rotavirus is noteworthy given the relative challenge of inactivating rotavirus with non-boiling HWT (as compared with other viral pathogens).^177^

Our results also show that reported boiling has a strong, and highly significant, protective effect for nonspecific diarrheal disease outcomes. To better contextualize these findings, in Table 2 we provide a comparison of the pooled OR for diarrheal outcomes associated with reported boiling and the pooled effect estimates from previous systematic reviews on diarrheal outcomes and other HWT methods (as mentioned earlier, most HWT health studies use nonspecific diarrhea as the primary outcome, so we cannot create similar tables to compare pathogen-specific outcomes). An important caveat, however, is that in contrast to most of these other systematic reviews, we did not restrict our inclusion criteria to include only experimental designs (i.e., those using randomized or quasi-randomized assignment and control groups), because there are no published reports of such studies for boiling. Therefore, it is likely that the effect estimates in Table 2 have lower likelihoods of bias as compared with our pooled estimate for reported boiling and diarrheal outcomes. In addition, the pooled estimate from our study does not control for safe post-boiling water storage (with consistent boiling adherence and safe storage, the protective effect might be stronger). With these caveats in mind, we see that the pooled effects associated with filtration are the strongest, followed closely by the pooled estimate for reported boiling from our study (based on data from seven studies). With regard to HWT methods and their impact on diarrheal outcomes, this side-by-side comparison suggests that boiling is at least as effective as the other primary methods of HWT, and perhaps more effective than some.

**Table 2:** Pooled effect estimates of HWT methods on diarrheal outcomes from other systematic review and meta analysis studies

HWT method	Pooled estimate	95% CI	Studies	Source
Boiling	OR = 0.58	0.45–0.77	7	This study
Chlorine	RR = 0.71	0.58–0.87	10	^13^
Chlorine	OR = 0.77	0.58–1.02	3	^30^
Chlorine	RR = 0.77	0.65–0.91	14	^28^
Filtration	OR = 0.37	0.27–0.49	2	^30^
Filtration	RR = 0.48	0.38–0.59	18	^28^
Filtration	RR = 0.53*	0.41–0.67	(∼14)†	^3^
Filtration: adjusted for non-blinding	RR = 0.66*	0.47–0.92	(∼14)†	^3^
Flocculant and disinfection	RR = 0.69	0.58–0.82	4	^28^
Flocculant and disinfection	OR = 0.77	0.65–0.90	2	^30^
Solar disinfection	RR = 0.62	0.42–0.94	4	^28^
Solar disinfection	OR = 0.69	0.63–0.74	2	^30^
Chlorine or solar disinfection‡	RR = 0.82*	0.69–0.96	(∼22)†	^3^
Chlorine or solar disinfection: adjusted‡	RR = 0.99*	0.76–1.27	(∼22)†	^3^
Various HWT	RR = 0.65	0.48–0.88	12	^29^
Various HWT	OR = 0.65	0.56–0.76	10	^30^
Various HWT	ES = 0.56§	0.48–0.65	28	^12^

CI = confidence interval; HWT = household water treatment; ES = effect size; OR = odds ratio; RR = risk ratio.

* The presented pooled effects from Wolf and others (2014) do not include studies/estimates with safe-storage.

† It was unclear from the text (or supplementary information) how many studies were used to derive these pooled estimates.

‡ The authors explained their decision to calculate the RR for chlorination and solar disinfection as follows: “The results for chlorine and solar interventions were very similar and so, for convenience, they were combined in all analyses” [p935].^3^

§ Waddington and others (2009) transformed study effect estimates into a “common metric” ES.

Our study had a number of limitations. The primary limitations were 2-fold: none of the included studies were based on experimental designs, and boiling was assessed via self-report in almost all studies, meaning there was likely substantial heterogeneity in boiling consistency and adherence. Indeed, there is likely substantial heterogeneity between (and within) studies due to differences in boiling methods, frequencies, durations, consistency of use, and methods for storing boiled water and associated risks of secondary contamination.^23,25,26^ Though the results we present here do not control for post-boiling safe storage (due to a lack of data), if we assume that many or most of the households from which data were collected did not practice safe post-boiling storage, boiling combined with safe storage would likely result in an even more preventative net effect for water-related infectious disease outcomes. For example, in Wolf and others’ systematic review,^3^ when the authors controlled for the use of safe storage, the pooled effect estimates for filtration and chlorine/solar disinfection were more protective (with and without adjustment for non-blinding).

Our study had other limitations as well. Among the 156 studies identified for full-text review, we were unable to retrieve the full-text for nine records, meaning potentially eligible data may not be included in our meta-analyses. Another limitation of our study (common to many such systematic reviews) is the treatment of reported RRs as ORs, because in cases where outcomes are not rare, ORs tend to be larger than RRs. In addition, as may be apparent from our assessment of study bias/quality, for a number of studies there were nontrivial differences in the apparent methodological rigor underlying data collection and analysis. In addition, six of the studies included in this meta-analysis were outbreak-motivated studies, meaning the effect associated with boiling might have been less pronounced during non-outbreak periods when the disease incidence and associated risks were lower. However, the potential bias associated with these outbreak investigations only had the potential to change our conclusions for the interpretation of reported boiling’s impact on bacterial outcomes (since four of the six outbreak studies focused on *V. cholera*, which we controlled for [see Figure 2]). Finally, the comparatively limited number of studies identified for some of the pathogen-specific outcomes makes it challenging to interpret many of the results, or to speak to the generalizability of our findings with regard to other populations and regions.

With regard to broader limitations, the current global estimates of boiling prevalence are mostly based on self-report, may be overreported in some instances, and do not provide sufficient data on differences in the consistency of boiling or on the use of safe or unsafe post-boiling storage. In addition, although many of the HWT RCT studies we identified and reviewed did mention the use of boiling in study control groups, none provided health outcome data for participants who practiced boiling (in the main text or online supplementary information). Similarly, in many of these and similar HWT-focused papers, baseline water treatment practices in the control group, such as boiling or filtration, are often aggregated into a catch-all category “water treatment.” Consequently, we were unable to extract data from many of the studies we identified as otherwise eligible (a point we sought to highlight in Figure 1). In the interests of improved reporting, replication, and facilitating systematic reviews, we therefore recommend that, when feasible, more comprehensive results and/or data from WASH RCTs should be provided in supplementary information and/or data repositories.

As mentioned earlier, the use of boiling in LMIC settings itself has a number of limitations: boiled water is susceptible to recontamination, boiling does not remove chemical or metal contaminants, the fuels needed for boiling can be relatively costly, and many of the fuels currently used to boil drinking water produce HAP. The first two limitations are, however, not unique to boiling. Solar and UV disinfection, as well as filtration, provide no residual disinfectant (and therefore require safe storage),^25^ and aside from flocculants and relatively expensive filters, none of the primary HWT methods adequately remove chemical or metal contaminants. In many LMIC settings, fuel costs may be a significant barrier to the adoption of boiling, and HAP is especially problematic in rural areas where households use wood, agricultural refuse, coal, or other solid-fuels to boil their water, as well as for cooking and heating. HAP exposure causes a number of cardiovascular and respiratory diseases, and is ranked eighth among global health risks.^7^ HAP exposure is also one of the primary environmental causes of premature death, with 3.9 million attributable deaths in 2010.^24^

As discussed earlier, unlike the variable effectiveness of other HWT methods, if drinking water is heated to boil, full pathogen inactivation should be achieved regardless of the organism groups, specific pathogens, or water turbidity. In light of the evidence of reported boiling’s impact on health outcomes presented here, and taking into consideration its widespread use globally and the well-documented challenges promoting retail HWT products,^11,12,15^ it may be worthwhile to evaluate the potential health gains that could be realized by building upon existing preferences for boiled water to promote safer and more reliable methods or technologies for water boiling. Such an effort would also require a clearer understanding of the sociocultural factors underlying preferences for boiling, as well as would-be barriers to adoption. In conclusion, we believe the evidence presented here highlights the need for a more proportionate focus on boiling in the WASH policy, practitioner, and research communities, and that a definitive boiling-focused RCT is justified.

CI = confidence interval; OR = odds ratio; R = rural; U = urban; k = 1,000. Cells with “–” indicate instances where there was too much collinearity with the associated covariate. Cells with “–” indicate instances where there were an insufficient number of observations available.

CI = confidence interval; OR = odds ratio; R = rural; U = urban; k = 1,000. Cells with “–” indicate instances where there was too much collinearity with the associated covariate. Cells with “–” indicate instances where there were an insufficient number of observations available

## Supplementary Material

Supplemental Figure and Table.
