# Supplementary material for: Effects of Boiling Drinking Water on Diarrhea and Pathogen-Specific Infections in Low- and Middle-Income Countries: A Systematic Review and Meta-Analysis
Source: Am J Trop Med Hyg. 2017 Sep 5;97(5):1362–77. doi: 10.4269/ajtmh.17-0190 (PMC5817760; doi:10.4269/ajtmh.17-0190)
Supplement: Supplementary file 1 [file tpmd170190.SD1.pdf]

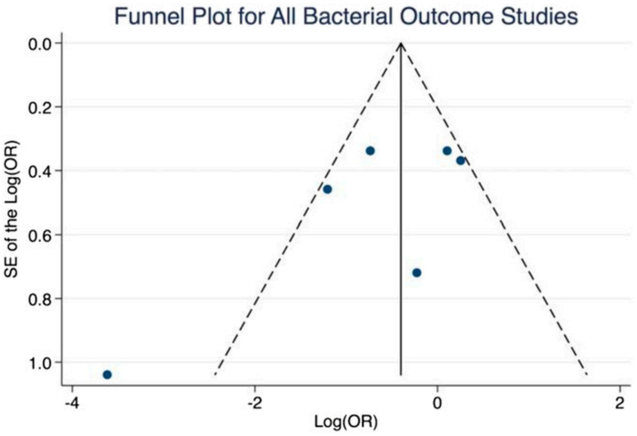

Supplemental Figure 1. Funnel plot for all bacterial outcome studies (using all study outcomes).

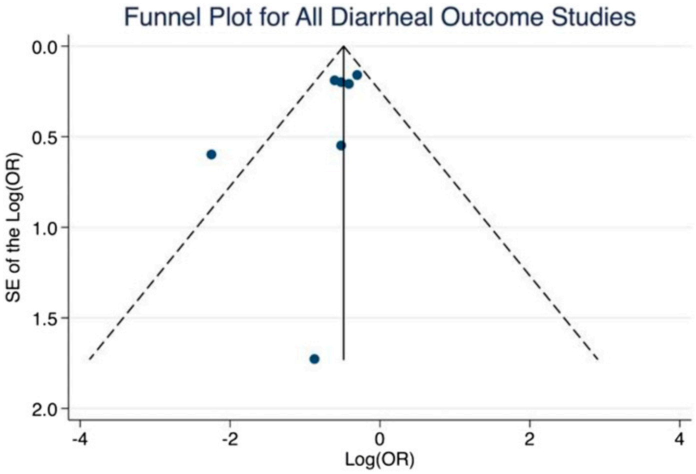

Supplemental Figure 2. Funnel plot for all diarrheal outcome studies (using all study outcomes).

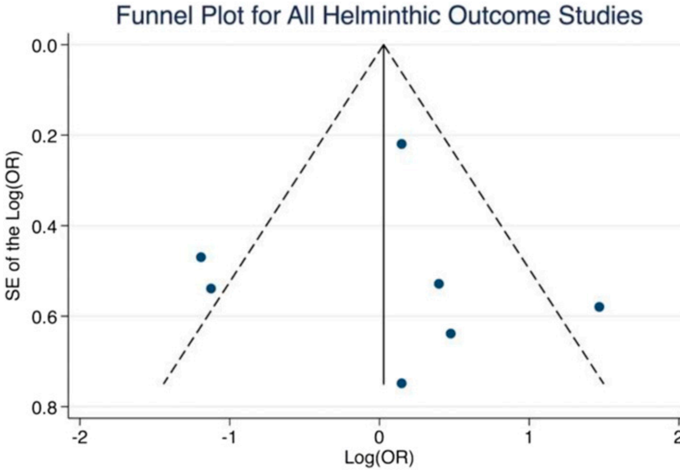

Supplemental Figure 3. Funnel plot for all helminthic outcom studies (using all study outcomes).

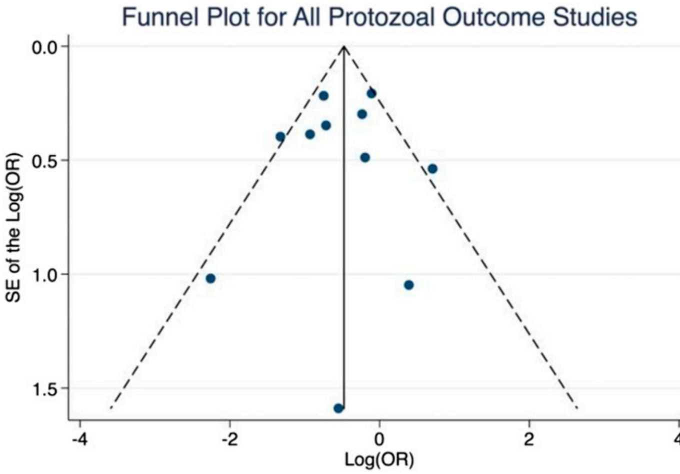

Supplemental Figure 4. Funnel plot for all protozoal outcom studies (using all study outcomes).

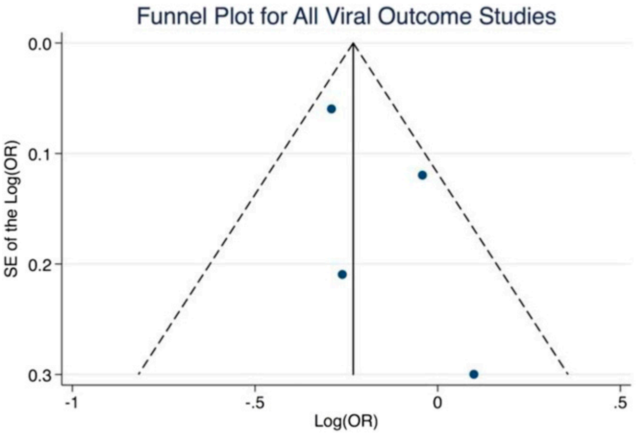

Supplemental Figure 5. Funnel plot for all viral outcome studies (using all study outcomes).

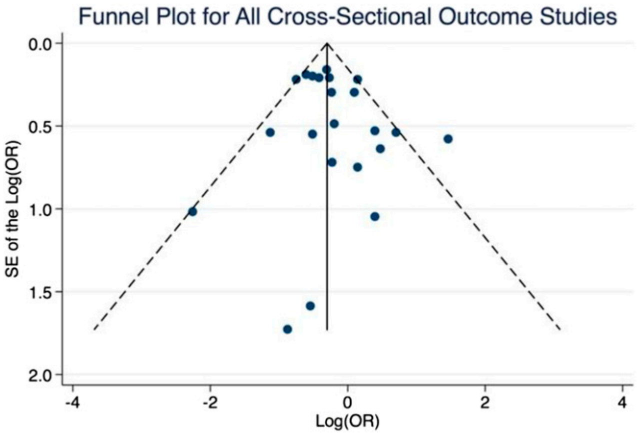

Supplemental Figure 7. Funnel plots for cross-sectional study outcomes.

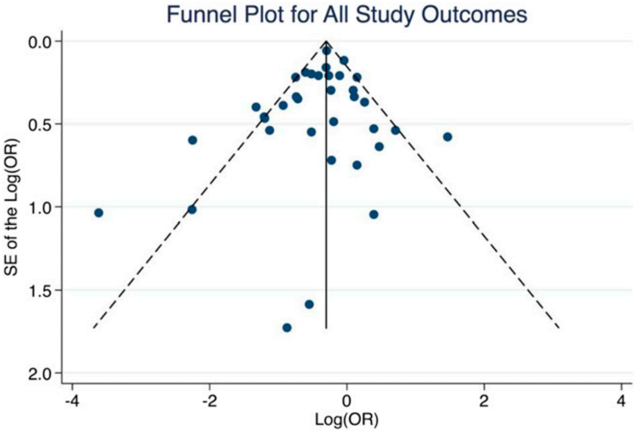

Supplemental Figure 6. Funnel plots for all study outcomes.

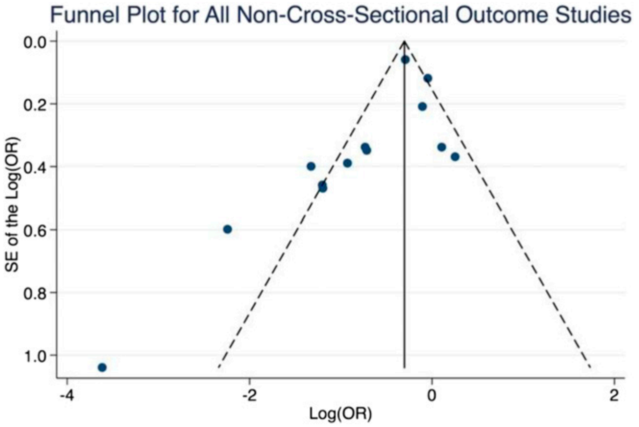

Supplemental Figure 8. Funnel plots for non-cross-sectional study outcomes.

Supplemental Table 1
Full list of search terms by search sets

1	2	3	4
Drinking water	Boiling and drinking water treatment	GI-related diseases	Low-/middle-income countries* (with "OR" inserted between names)
"Domestic water"	Boil	"Acute gastrointestinal"	Afghanistan OR Algeria OR Angola OR Anguilla OR Antigua OR Barbuda OR Argentina OR Armenia OR Armenian OR Aruba OR Azerbaijan OR Bahamas OR Bahrain OR Bangladesh OR Barbados OR Benin OR Byelarus OR Byelorussian OR Belarus OR Belorussian OR Belorussia OR Belize OR Bhutan OR Bolivia OR Botswana OR Brazil OR Brunei OR Burkina Faso OR "Burkina Fasso" OR "Upper Volta" OR Burundi OR Urundi OR Cambodia OR "Khmer Republic" OR Kampuchea OR Cameroon OR Cameroons OR Cameron OR Camerons OR "Cape Verde" OR "Cayman Islands" OR "Central African Republic" OR Chad OR Chile OR China OR Colombia OR Comoros OR "Comoro Islands" OR Comores OR Mayotte OR Congo OR Zaire OR "Cook Islands" OR "Costa Rica" OR "Cote dIvoire" OR "Ivory Coast" OR Croatia OR Cuba OR Cyprus OR Djibouti OR "French Somaliland" OR Dominica OR "Dominican Republic" OR "East Timor" OR "East Timur" OR "Timor Leste" OR Ecuador OR Egypt OR "United Arab Republic" OR "El Salvador" OR Eritrea OR Ethiopia OR "Falkland Islands" OR "Las Malvinas" OR Fiji OR Gabon OR "Gabonese Republic" OR Gambia OR Gaza OR "Georgia Republic" OR "Georgian Republic" OR Ghana OR "Gold Coast" OR Greece OR Grenada OR Guatemala OR Guinea OR Guam OR Guadeloupe OR Guiana OR Guyana OR Haiti OR Honduras OR "Hong Kong" OR India OR Maldives OR Indonesia OR Iran OR Iraq OR Jamaica OR Jordan OR Kazakhstan OR Kazakh OR Kenya OR Kiribati OR Korea OR Kosovo OR Kuwait OR Kyrgyzstan OR Kirghizia OR "Kyrgyz Republic" OR Kirghiz OR Kirgizstan OR "Lao PDR" OR Laos OR Lebanon OR Lesotho OR Basutoland OR Liberia OR Libya OR Macau OR Madagascar OR "Malagasy Republic" OR Maldives OR Malaysia OR Malaya OR Malay OR Sabah OR Sarawak OR Malawi OR Nyasaland OR Mali OR Malta OR "Marshall Islands" OR Martinique OR Mauritania OR Mauritius OR "Agalega Islands" OR Mexico OR Micronesia OR "Middle East" OR Mongolia OR Montserrat OR Morocco OR Ifni OR Mozambique OR Myanmar OR Myanma OR Burma OR Namibia OR Nauru OR Nepal OR Niui OR "Netherlands Antilles" OR "New Caledonia" OR Nicaragua OR Niger OR Nigeria OR "Northern Mariana Islands" OR Oman OR Mayotte OR Muscat OR Pakistan OR Palau OR Palestine OR Panama OR Paraguay OR Peru OR Philippines OR Philipines OR Phillipines OR Phillippines OR Polynesia OR
"Drinking water"	Boiled	"AGI"	
"Potable water"	Boiling	"AGII"	
"Tap water"	Decontamination	"HCGI"	
Water	Disinfect	"Highly credible gastrointestinal"	
"Water consumption"	Disinfectant	*Campylobacter*	
"Well water"	Disinfection	Cholera	
	Domestic	Cholerae	
	Household	Cryptosporidium	
	Inactivation	Diarrhea	
	Pasteurise	Diarrheal	
	Pasteurised	"Diarrheal disease"	
	Pasteurize	Diarrheoa	
	Pasteurized	Diarrheoal	
	"Point of use"	"Diarrheoal disease"	
	Point-of-use	Dysentery	
	"POU"	"E. coli"	
	Purification	Enteric	
	Purified	"Enteric virus"	
	Purify	"Enteric viruses"	
	Residential	Enterovirus	
	"Safe water"	Enteroviruses	
	Treat	"E. coli"	
	Treated	"Escherichia coli"	
	Treating	Gastrointestinal	
	Treatment	"Gastro intestinal"	
	"Water disinfection"	Gastro-enteric	
	"Water purification"	*Giardia*	
	"Water quality"	Helicobacter	
	"Water treatment"	Helminth	
		Hepatitis	
		Intestinal	
		Norovirus	
		"Norwold-like virus"	
		Rotavirus	
		Salmonella	
		Shigella	
		"Vibrio cholera"	
		"Vibrio cholerae"	
		Waterborne	
		Water-borne	
		"Waterborne pathogen"	
		"Waterborne pathogens"	

(continued)

Supplemental Table 1
Continued

1	2	3	4
Drinking water	Boiling and drinking water treatment	GI-related diseases	Low-/middle-income countries* (with "OR" inserted between names)
			"Puerto Rico" OR Qatar OR Reunion OR Rwanda OR Ruanda OR "Saint Kitts" OR "St Kitts" OR Nevis OR "Saint Lucia" OR "St Lucia" OR "Saint Vincent" OR "St Vincent" OR Grenadines OR Samoa OR "Samoan Islands" OR "Navigator Island" OR "Navigator Islands" OR "Sao Tome" OR "Saudi Arabia" OR Senegal OR Serbia OR Montenegro OR Seychelles OR "Sierra Leone" OR Singapore OR "Sri Lanka" OR Ceylon OR "Solomon Islands" OR Somalia OR "South Africa" OR Sudan OR Suriname OR Surinam OR Swaziland OR Syria OR Tajikistan OR Tadzhikistan OR Tadjikistan OR Tadzhik OR Tanzania OR Thailand OR Togo OR "Togolese Republic" OR Tokelau OR Tonga OR Trinidad OR Tobago OR Tunisia OR Turkey OR Turkmenistan OR Turkmen OR "Turks Caicos" OR "Tuvalu Uganda" OR "United Arab Emirates" OR Uruguay OR Uzbekistan OR Uzbek OR Vanuatu OR "New Hebrides" OR Venezuela OR Vietnam OR "Viet Nam" OR "Virgin Islands" OR "West Bank" OR Yemen OR Yugoslavia OR Zambia OR Zimbabwe

Boolean operators: "OR" between row terms in each column, "AND" between full columns.
* This list was adapted from Bain and others.[2]

SUPPLEMENTAL TABLE 2

Search terms, notes, and results for PubMed (MEDLINE and other sources)

Boolean operators used between search term sets	Database searched (date of search)	Fields searched (limitations)
1 AND 2 AND 3 AND 4	PubMed (Jan 21, 2016) https://www.ncbi.nlm.nih.gov/pubmed/	Title/Abstract, MeSH Major Topics (none) [only “Title/Abstract” for set #4]

Results	Search text
1,306	((((((“Domestic water”[Title/Abstract] OR “Drinking water”[Title/Abstract] OR “Potable water”[Title/Abstract] OR “Tap water”[Title/Abstract] OR “Water consumption”[Title/Abstract] OR “Well water”[Title/Abstract])) OR (“Domestic water” OR “Drinking water” OR “Potable water” OR “Tap water” OR “Water consumption” OR “Well water”[MeSH Major Topic]))) AND (((Boil[Title/Abstract] OR Boiled[Title/Abstract] OR Boiling[Title/Abstract] OR Decontamination[Title/Abstract] OR Disinfect[Title/Abstract] OR Disinfectant[Title/Abstract] OR Disinfection[Title/Abstract] OR Domestic[Title/Abstract] OR Household[Title/Abstract] OR Inactivation[Title/Abstract] OR Pasteurise[Title/Abstract] OR Pasteurised[Title/Abstract] OR Pasteurize[Title/Abstract] OR Pasteurized[Title/Abstract] OR “Point of use”[Title/Abstract] OR Point-of-use[Title/Abstract] OR “POU”[Title/Abstract] OR Purification[Title/Abstract] OR Purified[Title/Abstract] OR Purify[Title/Abstract] OR Residential[Title/Abstract] OR “Safe water”[Title/Abstract] OR Treat[Title/Abstract] OR Treated[Title/Abstract] OR Treating[Title/Abstract] OR Treatment[Title/Abstract] OR “Water disinfection”[Title/Abstract] OR “Water purification”[Title/Abstract] OR “Water quality”[Title/Abstract] OR “Water treatment”[Title/Abstract])) OR (Boil OR Boiled OR Boiling OR Decontamination OR Disinfect OR Disinfectant OR Disinfection OR Domestic OR Household OR Inactivation OR Pasteurise OR Pasteurised OR Pasteurize OR Pasteurized OR “Point of use” OR Point-of-use OR “POU” OR Purification OR Purified OR Purify OR Residential OR “Safe water” OR Treat OR Treated OR Treating OR Treatment OR “Water disinfection” OR “Water purification” OR “Water quality” OR “Water treatment”[MeSH Major Topic]))) AND (((“Acute gastrointestinal”[Title/Abstract] OR “AGI” [Title/Abstract] OR “AGII” [Title/Abstract] OR “HCGI” [Title/Abstract] OR “Highly credible gastrointestinal” [Title/Abstract] OR *Campylobacter*[Title/Abstract] OR Cholera[Title/Abstract] OR Cholerae[Title/Abstract] OR Cryptosporidium[Title/Abstract] OR Diarrhea[Title/Abstract] OR Diarrheal[Title/Abstract] OR “Diarrheal disease” [Title/Abstract] OR Diarrheoa[Title/Abstract] OR Diarrheoal[Title/Abstract] OR “Diarrheoal disease” [Title/Abstract] OR Dysentery[Title/Abstract] OR “E. coli” [Title/Abstract] OR Enteric[Title/Abstract] OR “Enteric virus” [Title/Abstract] OR “Enteric viruses” [Title/Abstract] OR Enterovirus[Title/Abstract] OR Enteroviruses[Title/Abstract] OR “E. coli” [Title/Abstract] OR “Escherichia coli” [Title/Abstract] OR Gastrointestinal[Title/Abstract] OR “Gastro intestinal” [Title/Abstract] OR Gastro-enteric[Title/Abstract] OR *Giardia*[Title/Abstract] OR Helicobacter[Title/Abstract] OR Helminth [Title/Abstract] OR Hepatitis[Title/Abstract] OR Intestinal[Title/Abstract] OR Norovirus[Title/Abstract] OR “Norwold-like virus” [Title/Abstract] OR Rotavirus[Title/Abstract] OR *Salmonella*[Title/Abstract] OR Shigella[Title/Abstract] OR “Vibrio cholera” [Title/Abstract] OR “Vibrio cholerae” [Title/Abstract] OR Waterborne[Title/Abstract] OR Water-borne[Title/Abstract] OR “Waterborne pathogen” [Title/Abstract] OR “Waterborne pathogens”[Title/Abstract])) OR (“Acute gastrointestinal” OR “AGI” OR “AGII” OR “HCGI” OR “Highly credible gastrointestinal” OR *Campylobacter* OR Cholera OR Cholerae OR Cryptosporidium OR Diarrhea OR Diarrheal OR “Diarrheal disease” OR Diarrheoa OR Diarrheoal OR “Diarrheoal disease” OR Dysentery OR “E. coli” OR Enteric OR “Enteric virus” OR “Enteric viruses” OR Enterovirus OR Enteroviruses OR “E. coli” OR “Escherichia coli” OR Gastrointestinal OR “Gastro intestinal” OR Gastro-enteric OR *Giardia* OR Helicobacter OR Helminth OR Hepatitis OR Intestinal OR Norovirus OR “Norwold-like virus” OR Rotavirus OR *Salmonella* OR Shigella OR “Vibrio cholera” OR “Vibrio cholerae” OR Waterborne OR Water-borne OR “Waterborne pathogen” OR “Waterborne pathogens”[MeSH Major Topic]))) AND ((Afghanistan[Title/Abstract] OR Algeria[Title/Abstract] OR Angola[Title/Abstract] OR Anguilla[Title/Abstract] OR Antigua[Title/Abstract] OR Barbuda[Title/Abstract] OR Argentina[Title/Abstract] OR Armenia[Title/Abstract] OR Armenian[Title/Abstract] OR Aruba[Title/Abstract] OR Azerbaijan[Title/Abstract] OR Bahamas[Title/Abstract] OR Bahrain[Title/Abstract] OR Bangladesh[Title/Abstract] OR Barbados[Title/Abstract] OR Benin[Title/Abstract] OR Byelarus[Title/Abstract] OR Byelorussian[Title/Abstract] OR Belarus[Title/Abstract] OR Belorussian[Title/Abstract] OR Belorussia[Title/Abstract] OR Belize[Title/Abstract] OR Bhutan [Title/Abstract] OR Bolivia[Title/Abstract] OR Botswana[Title/Abstract] OR Brazil[Title/Abstract] OR Brunei[Title/Abstract] OR Burkina Faso[Title/Abstract] OR “Burkina Fasso”[Title/Abstract] OR “Upper Volta”[Title/Abstract] OR Burundi[Title/Abstract] OR Urundi[Title/Abstract] OR Cambodia[Title/Abstract] OR “Khmer Republic”[Title/Abstract] OR Kampuchea[Title/Abstract] OR Cameroon[Title/Abstract] OR Cameroons[Title/Abstract] OR Cameron[Title/Abstract] OR Camerons[Title/Abstract] OR “Cape Verde”[Title/Abstract] OR “Cayman Islands”[Title/Abstract] OR “Central African Republic”[Title/Abstract] OR Chad[Title/Abstract] OR Chile[Title/Abstract] OR China[Title/Abstract] OR Colombia[Title/Abstract] OR Comoros[Title/Abstract] OR “Comoro Islands”[Title/Abstract] OR Comores[Title/Abstract] OR Mayotte [Title/Abstract] OR Congo[Title/Abstract] OR Zaire[Title/Abstract] OR “Cook Islands”[Title/Abstract] OR “Costa Rica”[Title/Abstract] OR “Cote dIvoire”[Title/Abstract] OR “Ivory Coast”[Title/Abstract] OR Croatia[Title/Abstract] OR Cuba[Title/Abstract] OR Cyprus[Title/Abstract] OR Djibouti[Title/Abstract] OR “French Somaliland”[Title/Abstract] OR Dominica[Title/Abstract] OR “Dominican Republic”[Title/Abstract] OR “East Timor”[Title/Abstract] OR “East Timur”[Title/Abstract] OR “Timor Leste”[Title/Abstract] OR Ecuador[Title/Abstract] OR Egypt[Title/Abstract] OR “United Arab Republic”[Title/Abstract] OR “El Salvador”[Title/Abstract] OR Eritrea[Title/Abstract] OR Ethiopia[Title/Abstract] OR “Falkland Islands”[Title/Abstract] OR “Las Malvinas”[Title/Abstract] OR Fiji[Title/Abstract] OR Gabon[Title/Abstract] OR “Gabonese Republic”[Title/Abstract] OR Gambia[Title/Abstract] OR Gaza[Title/Abstract] OR “Georgia Republic”[Title/Abstract] OR “Georgian Republic”[Title/Abstract] OR Ghana[Title/Abstract] OR “Gold Coast”[Title/Abstract] OR Greece [Title/Abstract] OR Grenada[Title/Abstract] OR Guatemala[Title/Abstract] OR Guinea[Title/Abstract] OR Guam[Title/Abstract] OR Guadeloupe[Title/Abstract] OR Guiana[Title/Abstract] OR Guyana[Title/Abstract] OR Haiti[Title/Abstract] OR Honduras[Title/Abstract] OR “Hong Kong”[Title/Abstract] OR India[Title/Abstract] OR Maldives[Title/Abstract] OR Indonesia[Title/Abstract] OR Iran[Title/Abstract] OR Iraq[Title/Abstract] OR Jamaica[Title/Abstract] OR Jordan[Title/Abstract] OR Kazakhstan[Title/Abstract] OR Kazakh[Title/Abstract] OR Kenya[Title/Abstract] OR Kiribati[Title/Abstract] OR Korea[Title/Abstract] OR Kosovo[Title/Abstract] OR Kuwait[Title/Abstract] OR Kyrgyzstan[Title/Abstract] OR Kirghizia[Title/Abstract] OR “Kyrgyz Republic”[Title/Abstract] OR Kirghiz[Title/Abstract] OR Kirgizstan[Title/Abstract] OR “Lao PDR”[Title/Abstract] OR Laos[Title/Abstract] OR Lebanon[Title/Abstract] OR Lesotho[Title/Abstract] OR Basutoland[Title/Abstract] OR Liberia[Title/Abstract] OR Libya[Title/Abstract] OR Macau[Title/Abstract] OR Madagascar [Title/Abstract] OR “Malagasy Republic”[Title/Abstract] OR Maldives[Title/Abstract] OR Malaysia[Title/Abstract] OR

(continued)

Supplemental Table 2

Continued

Boolean operators used between search term sets	Database searched (date of search)	Fields searched (limitations)
1 AND 2 AND 3 AND 4	PubMed (Jan 21, 2016) https://www.ncbi.nlm.nih.gov/pubmed/	Title/Abstract, MeSH Major Topics (none) [only "Title/Abstract" for set #4]

Results	Search text
	Malaya[Title/Abstract] OR Malay[Title/Abstract] OR Sabah[Title/Abstract] OR Sarawak[Title/Abstract] OR Malawi[Title/Abstract] OR Nyasaland[Title/Abstract] OR Mali[Title/Abstract] OR Malta[Title/Abstract] OR "Marshall Islands"[Title/Abstract] OR Martinique[Title/Abstract] OR Mauritania[Title/Abstract] OR Mauritius[Title/Abstract] OR "Agalega Islands"[Title/Abstract] OR Mexico[Title/Abstract] OR Micronesia[Title/Abstract] OR "Middle East"[Title/Abstract] OR Mongolia[Title/Abstract] OR Montserrat[Title/Abstract] OR Morocco[Title/Abstract] OR Ifni[Title/Abstract] OR Mozambique[Title/Abstract] OR Myanmar[Title/Abstract] OR Myanma[Title/Abstract] OR Burma[Title/Abstract] OR Namibia[Title/Abstract] OR Nauru[Title/Abstract] OR Nepal[Title/Abstract] OR Niui[Title/Abstract] OR "Netherlands Antilles"[Title/Abstract] OR "New Caledonia"[Title/Abstract] OR Nicaragua[Title/Abstract] OR Niger[Title/Abstract] OR Nigeria[Title/Abstract] OR "Northern Mariana Islands"[Title/Abstract] OR Oman[Title/Abstract] OR Mayotte[Title/Abstract] OR Muscat[Title/Abstract] OR Pakistan[Title/Abstract] OR Palau[Title/Abstract] OR Palestine[Title/Abstract] OR Panama[Title/Abstract] OR Paraguay[Title/Abstract] OR Peru[Title/Abstract] OR Philippines[Title/Abstract] OR Philipines[Title/Abstract] OR Phillipines[Title/Abstract] OR Phillippines[Title/Abstract] OR Polynesia[Title/Abstract] OR "Puerto Rico"[Title/Abstract] OR Qatar[Title/Abstract] OR Reunion[Title/Abstract] OR Rwanda[Title/Abstract] OR Ruanda[Title/Abstract] OR "Saint Kitts"[Title/Abstract] OR "St Kitts"[Title/Abstract] OR Nevis[Title/Abstract] OR "Saint Lucia"[Title/Abstract] OR "St Lucia"[Title/Abstract] OR "Saint Vincent"[Title/Abstract] OR "St Vincent"[Title/Abstract] OR Grenadines[Title/Abstract] OR Samoa[Title/Abstract] OR "Samoan Islands"[Title/Abstract] OR "Navigator Island"[Title/Abstract] OR "Navigator Islands"[Title/Abstract] OR "Sao Tome"[Title/Abstract] OR "Saudi Arabia"[Title/Abstract] OR Senegal[Title/Abstract] OR Serbia[Title/Abstract] OR Montenegro[Title/Abstract] OR Seychelles[Title/Abstract] OR "Sierra Leone"[Title/Abstract] OR Singapore[Title/Abstract] OR "Sri Lanka"[Title/Abstract] OR Ceylon[Title/Abstract] OR "Solomon Islands"[Title/Abstract] OR Somalia[Title/Abstract] OR "South Africa"[Title/Abstract] OR Sudan[Title/Abstract] OR Suriname[Title/Abstract] OR Surinam[Title/Abstract] OR Swaziland[Title/Abstract] OR Syria[Title/Abstract] OR Tajikistan[Title/Abstract] OR Tadzhikistan[Title/Abstract] OR Tadjikistan[Title/Abstract] OR Tadzhik[Title/Abstract] OR Tanzania[Title/Abstract] OR Thailand[Title/Abstract] OR Togo[Title/Abstract] OR "Togolese Republic"[Title/Abstract] OR Tokelau[Title/Abstract] OR Tonga[Title/Abstract] OR Trinidad[Title/Abstract] OR Tobago[Title/Abstract] OR Tunisia[Title/Abstract] OR Turkey[Title/Abstract] OR Turkmenistan[Title/Abstract] OR Turkmen[Title/Abstract] OR "Turks Caicos"[Title/Abstract] OR "Tuvalu Uganda"[Title/Abstract] OR "United Arab Emirates"[Title/Abstract] OR Uruguay[Title/Abstract] OR Uzbekistan[Title/Abstract] OR Uzbek[Title/Abstract] OR Vanuatu[Title/Abstract] OR "New Hebrides"[Title/Abstract] OR Venezuela[Title/Abstract] OR Vietnam[Title/Abstract] OR "Viet Nam"[Title/Abstract] OR "Virgin Islands"[Title/Abstract] OR "West Bank"[Title/Abstract] OR Yemen[Title/Abstract] OR Yugoslavia[Title/Abstract] OR Zambia[Title/Abstract] OR Zimbabwe[Title/Abstract]))

Supplemental Table 3

Search terms, notes, and results for EMBASE (and EMBASE Classic)

Boolean operators used between search term sets	Database searched (date of search)	Fields searched (limitations)
1 AND 2 AND 3 AND 4	EMBASE (Jan 21, 2016) https://www.elsevier.com/solutions/embase-biomedical-research	Abstracts, Titles, Index terms (none)

Results	Search text
1,329	('domestic water':de,ab,ti OR 'drinking water':de,ab,ti OR 'potable water':de,ab,ti OR 'tap water':de,ab,ti OR 'water consumption':de,ab,ti OR 'well water':de,ab,ti) AND (boil:de,ab,ti OR boiled:de,ab,ti OR boiling:de,ab,ti OR decontamination:de,ab,ti OR disinfect:de,ab,ti OR disinfectant:de,ab,ti OR disinfection:de,ab,ti OR domestic:de,ab,ti OR household:de,ab,ti OR inactivation:de,ab,ti OR pasteurise:de,ab,ti OR pasteurised:de,ab,ti OR pasteurize:de,ab,ti OR pasteurized:de,ab,ti OR 'point of use':de,ab,ti OR 'pou':de,ab,ti OR purification:de,ab,ti OR purified:de,ab,ti OR purify:de,ab,ti OR residential:de,ab,ti OR 'safe water':de,ab,ti OR treat:de,ab,ti OR treated:de,ab,ti OR treating:de,ab,ti OR treatment:de,ab,ti OR 'water disinfection':de,ab,ti OR 'water purification':de,ab,ti OR 'water quality':de,ab,ti OR 'water treatment':de,ab,ti) AND ('acute gastrointestinal':de,ab,ti OR 'agi':de,ab,ti OR 'agii':de,ab,ti OR 'hcgi':de,ab,ti OR 'highly credible gastrointestinal':de,ab,ti OR *Campylobacter*:de,ab,ti OR cholera:de,ab,ti OR cholerae:de,ab,ti OR cryptosporidium:de,ab,ti OR diarrhea:de,ab,ti OR diarrheal:de,ab,ti OR 'diarrheal disease':de,ab,ti OR diarrheoa:de,ab,ti OR diarrheoal:de,ab,ti OR 'diarrheoal disease':de,ab,ti OR dysentery:de,ab,ti OR enteric:de,ab,ti OR 'enteric virus':de,ab,ti OR 'enteric viruses':de,ab,ti OR enterovirus:de,ab,ti OR enteroviruses:de,ab,ti OR '*E. coli*':de,ab,ti OR '*Escherichia coli*':de,ab,ti OR gastrointestinal:de,ab,ti OR 'gastro intestinal':de,ab,ti OR 'gastro enteric':de,ab,ti OR *Giardia*:de,ab,ti OR helicobacter:de,ab,ti OR helminth:de,ab,ti OR hepatitis:de,ab,ti OR intestinal:de,ab,ti OR norovirus:de,ab,ti OR 'norwold-like virus':de,ab,ti OR rotavirus:de,ab,ti OR *Salmonella*:de,ab,ti OR shigella:de,ab,ti OR 'vibrio cholera':de,ab,ti OR 'vibrio cholerae':de,ab,ti OR waterborne:de,ab,ti OR 'water borne':de,ab,ti OR 'waterborne pathogen':de,ab,ti OR 'waterborne pathogens':de,ab,ti) AND (afghanistan:de,ab,ti OR algeria:de,ab,ti OR angola:de,ab,ti OR anguilla:de,ab,ti OR antigua:de,ab,ti OR barbuda:de,ab,ti OR argentina:de,ab,ti OR armenia:de,ab,ti OR armenian:de,ab,ti OR aruba:de,ab,ti OR azerbaijan:de,ab,ti OR bahamas:de,ab,ti OR bahrain:de,ab,ti OR bangladesh:de,ab,ti OR barbados:de,ab,ti OR benin:de,ab,ti OR byelarus:de,ab,ti OR byelorussian:de,ab,ti OR belarus:de,ab,ti OR belorussian:de,ab,ti OR belorussia:de,ab,ti OR belize:de,ab,ti OR bhutan:de,ab,ti OR bolivia:de,ab,ti OR botswana:de,ab,ti OR brazil:de,ab,ti OR brunei:de,ab,ti OR burkina:de,ab,ti AND faso:de,ab,ti OR 'burkina fasso':de,ab,ti OR 'upper volta':de,ab,ti OR burundi:de,ab,ti OR urundi:de,ab,ti OR cambodia:de,ab,ti OR 'khmer republic':de,ab,ti OR kampuchea:de,ab,ti OR cameroon:de,ab,ti OR cameroons:de,ab,ti OR cameron:de,ab,ti OR camerons:de,ab,ti OR 'cape verde':de,ab,ti OR 'cayman islands':de,ab,ti OR 'central african republic':de,ab,ti OR chad:de,ab,ti OR chile:de,ab,ti OR china:de,ab,ti OR colombia:de,ab,ti OR comoros:de,ab,ti OR 'comoro islands':de,ab,ti OR comores:de,ab,ti OR congo:de,ab,ti OR zaire:de,ab,ti OR 'cook islands':de,ab,ti OR 'costa rica':de,ab,ti OR 'cote divoire':de,ab,ti OR 'ivory coast':de,ab,ti OR croatia:de,ab,ti OR cuba:de,ab,ti OR cyprus:de,ab,ti OR djibouti:de,ab,ti OR 'french somaliland':de,ab,ti OR dominica:de,ab,ti OR 'dominican republic':de,ab,ti OR 'east timor':de,ab,ti OR 'east timur':de,ab,ti OR 'timor leste':de,ab,ti OR ecuador:de,ab,ti OR egypt:de,ab,ti OR 'united arab republic':de,ab,ti OR 'el salvador':de,ab,ti OR eritrea:de,ab,ti OR ethiopia:de,ab,ti OR 'falkland islands':de,ab,ti OR 'las malvinas':de,ab,ti OR fiji:de,ab,ti OR gabon:de,ab,ti OR 'gabonese republic':de,ab,ti OR gambia:de,ab,ti OR gaza:de,ab,ti OR 'georgia republic':de,ab,ti OR 'georgian republic':de,ab,ti OR ghana:de,ab,ti OR 'gold coast':de,ab,ti OR greece:de,ab,ti OR grenada:de,ab,ti OR guatemala:de,ab,ti OR guinea:de,ab,ti OR guam:de,ab,ti OR guadeloupe:de,ab,ti OR guiana:de,ab,ti OR guyana:de,ab,ti OR haiti:de,ab,ti OR honduras:de,ab,ti OR 'hong kong':de,ab,ti OR india:de,ab,ti OR indonesia:de,ab,ti OR iran:de,ab,ti OR iraq:de,ab,ti OR jamaica:de,ab,ti OR jordan:de,ab,ti OR kazakhstan:de,ab,ti OR kazakh:de,ab,ti OR kenya:de,ab,ti OR kiribati:de,ab,ti OR korea:de,ab,ti OR kosovo:de,ab,ti OR kuwait:de,ab,ti OR kyrgyzstan:de,ab,ti OR kirghizia:de,ab,ti OR 'kyrgyz republic':de,ab,ti OR kirghiz:de,ab,ti OR kirgizstan:de,ab,ti OR 'lao pdr':de,ab,ti OR laos:de,ab,ti OR lebanon:de,ab,ti OR lesotho:de,ab,ti OR basutoland:de,ab,ti OR liberia:de,ab,ti OR libya:de,ab,ti OR macau:de,ab,ti OR madagascar:de,ab,ti OR 'malagasy republic':de,ab,ti OR maldives:de,ab,ti OR malaysia:de,ab,ti OR malaya:de,ab,ti OR malay:de,ab,ti OR sabah:de,ab,ti OR sarawak:de,ab,ti OR malawi:de,ab,ti OR nyasaland:de,ab,ti OR mali:de,ab,ti OR malta:de,ab,ti OR 'marshall islands':de,ab,ti OR martinique:de,ab,ti OR mauritania:de,ab,ti OR mauritius:de,ab,ti OR 'agalega islands':de,ab,ti OR mexico:de,ab,ti OR micronesia:de,ab,ti OR 'middle east':de,ab,ti OR mongolia:de,ab,ti OR montserrat:de,ab,ti OR morocco:de,ab,ti OR ifni:de,ab,ti OR mozambique:de,ab,ti OR myanmar:de,ab,ti OR myanma:de,ab,ti OR burma:de,ab,ti OR namibia:de,ab,ti OR nauru:de,ab,ti OR nepal:de,ab,ti OR niui:de,ab,ti OR 'netherlands antilles':de,ab,ti OR 'new caledonia':de,ab,ti OR nicaragua:de,ab,ti OR niger:de,ab,ti OR nigeria:de,ab,ti OR 'northern mariana islands':de,ab,ti OR oman:de,ab,ti OR mayotte:de,ab,ti OR muscat:de,ab,ti OR pakistan:de,ab,ti OR palau:de,ab,ti OR palestine:de,ab,ti OR panama:de,ab,ti OR paraguay:de,ab,ti OR peru:de,ab,ti OR philippines:de,ab,ti OR philipines:de,ab,ti OR phillipines:de,ab,ti OR phillippines:de,ab,ti OR polynesia:de,ab,ti OR 'puerto rico':de,ab,ti OR qatar:de,ab,ti OR reunion:de,ab,ti OR rwanda:de,ab,ti OR ruanda:de,ab,ti OR 'saint kitts':de,ab,ti OR 'st kitts':de,ab,ti OR nevis:de,ab,ti OR 'saint lucia':de,ab,ti OR 'st lucia':de,ab,ti OR 'saint vincent':de,ab,ti OR 'st vincent':de,ab,ti OR grenadines:de,ab,ti OR samoa:de,ab,ti OR 'samoan islands':de,ab,ti OR 'navigator island':de,ab,ti OR 'navigator islands':de,ab,ti OR 'sao tome':de,ab,ti OR 'saudi arabia':de,ab,ti OR senegal:de,ab,ti OR montenegro:de,ab,ti OR serbia:de,ab,ti OR seychelles:de,ab,ti OR 'sierra leone':de,ab,ti OR singapore:de,ab,ti OR 'sri lanka':de,ab,ti OR ceylon:de,ab,ti OR 'solomon islands':de,ab,ti OR somalia:de,ab,ti OR 'south africa':de,ab,ti OR sudan:de,ab,ti OR suriname:de,ab,ti OR surinam:de,ab,ti OR swaziland:de,ab,ti OR syria:de,ab,ti OR tajikistan:de,ab,ti OR tadzhikistan:de,ab,ti OR tadjikistan:de,ab,ti OR tadzhik:de,ab,ti OR tanzania:de,ab,ti OR thailand:de,ab,ti OR togo:de,ab,ti OR 'togolese republic':de,ab,ti OR tokelau:de,ab,ti OR tonga:de,ab,ti OR trinidad:de,ab,ti OR tobago:de,ab,ti OR tunisia:de,ab,ti OR turkey:de,ab,ti OR turkmenistan:de,ab,ti OR turkmen:de,ab,ti OR 'turks caicos':de,ab,ti OR 'tuvalu uganda':de,ab,ti OR 'united arab emirates':de,ab,ti OR uruguay:de,ab,ti OR uzbekistan:de,ab,ti OR uzbek:de,ab,ti OR vanuatu:de,ab,ti OR 'new hebrides':de,ab,ti OR venezuela:de,ab,ti OR vietnam:de,ab,ti OR 'viet nam':de,ab,ti OR 'virgin islands':de,ab,ti OR 'west bank':de,ab,ti OR yemen:de,ab,ti OR yugoslavia:de,ab,ti OR zambia:de,ab,ti OR zimbabwe:de,ab,ti)

Supplemental Table 4

Search terms, notes, and results for Web of Science

Boolean operators used between search-term sets	Database searched (date of search)	Fields searched (limitations)
1 AND 2 AND 3 AND 4	Web of Science (Jan 21, 2016) http://apps.webofknowledge.com/ [Web of Science database only]	Topic (none) For search set #4: Title

Results	Search text
1,270	(TS=("Domestic water" OR "Drinking water" OR "Potable water" OR "Tap water" OR "Water consumption" OR "Well water")) AND (TS=(Boil OR Boiled OR Boiling OR Decontamination OR Disinfect OR Disinfectant OR Disinfection OR Domestic OR Household OR Inactivation OR Pasteurise OR Pasteurised OR Pasteurize OR Pasteurized OR "Point of use" OR Point-of-use OR "POU" OR Purification OR Purified OR Purify OR Residential OR "Safe water" OR Treat OR Treated OR Treating OR Treatment OR "Water disinfection" OR "Water purification" OR "Water quality" OR "Water treatment")) AND (TS=(Acute gastrointestinal OR AGI OR AGII OR HCGI OR Highly credible gastrointestinal OR *Campylobacter* OR Cholera OR Cholerae OR Cryptosporidium OR Diarrhea OR Diarrheal OR Diarrheal disease OR Diarrheoa OR Diarrheoal OR Diarrheoal disease OR Dysentery OR *E. coli* OR Enteric OR Enteric virus OR Enteric viruses OR Enterovirus OR Enteroviruses OR *E. coli* OR *Escherichia coli* OR Gastrointestinal OR Gastro intestinal OR Gastro-enteric OR *Giardia* OR Helicobacter OR Helminth OR Hepatitis OR Intestinal OR Norovirus OR Norwold-like virus OR Rotavirus OR *Salmonella* OR Shigella OR Vibrio cholera OR Vibrio cholerae OR Waterborne OR Water-borne OR Waterborne pathogen OR Waterborne pathogens)) AND (TI=(Afghanistan OR Algeria OR Angola OR Anguilla OR Antigua OR Barbuda OR Argentina OR Armenia OR Armenian OR Aruba OR Azerbaijan OR Bahamas OR Bahrain OR Bangladesh OR Barbados OR Benin OR Byelarus OR Byelorussian OR Belarus OR Belorussian OR Belorussia OR Belize OR Bhutan OR Bolivia OR Botswana OR Brazil OR Brunei OR Burkina Faso OR "Burkina Fasso" OR "Upper Volta" OR Burundi OR Urundi OR Cambodia OR "Khmer Republic" OR Kampuchea OR Cameroon OR Cameroons OR Cameron OR Camerons OR "Cape Verde" OR "Cayman Islands" OR "Central African Republic" OR Chad OR Chile OR China OR Colombia OR Comoros OR "Comoro Islands" OR Comores OR Mayotte OR Congo OR Zaire OR "Cook Islands" OR "Costa Rica" OR "Cote dIvoire" OR "Ivory Coast" OR Croatia OR Cuba OR Cyprus OR Djibouti OR "French Somaliland" OR Dominica OR "Dominican Republic" OR "East Timor" OR "East Timur" OR "Timor Leste" OR Ecuador OR Egypt OR "United Arab Republic" OR "El Salvador" OR Eritrea OR Ethiopia OR "Falkland Islands" OR "Las Malvinas" OR Fiji OR Gabon OR "Gabonese Republic" OR Gambia OR Gaza OR "Georgia Republic" OR "Georgian Republic" OR Ghana OR "Gold Coast" OR Greece OR Grenada OR Guatemala OR Guinea OR Guam OR Guadeloupe OR Guiana OR Guyana OR Haiti OR Honduras OR "Hong Kong" OR India OR Maldives OR Indonesia OR Iran OR Iraq OR Jamaica OR Jordan OR Kazakhstan OR Kazakh OR Kenya OR Kiribati OR Korea OR Kosovo OR Kuwait OR Kyrgyzstan OR Kirghizia OR "Kyrgyz Republic" OR Kirghiz OR Kirgizstan OR "Lao PDR" OR Laos OR Lebanon OR Lesotho OR Basutoland OR Liberia OR Libya OR Macau OR Madagascar OR "Malagasy Republic" OR Maldives OR Malaysia OR Malaya OR Malay OR Sabah OR Sarawak OR Malawi OR Nyasaland OR Mali OR Malta OR "Marshall Islands" OR Martinique OR Mauritania OR Mauritius OR "Agalega Islands" OR Mexico OR Micronesia OR "Middle East" OR Mongolia OR Montserrat OR Morocco OR Ifni OR Mozambique OR Myanmar OR Myanma OR Burma OR Namibia OR Nauru OR Nepal OR Niui OR "Netherlands Antilles" OR "New Caledonia" OR Nicaragua OR Niger OR Nigeria OR "Northern Mariana Islands" OR Oman OR Mayotte OR Muscat OR Pakistan OR Palau OR Palestine OR Panama OR Paraguay OR Peru OR Philippines OR Philipines OR Phillipines OR Phillippines OR Polynesia OR "Puerto Rico" OR Qatar OR Reunion OR Rwanda OR Ruanda OR "Saint Kitts" OR "St Kitts" OR Nevis OR "Saint Lucia" OR "St Lucia" OR "Saint Vincent" OR "St Vincent" OR Grenadines OR Samoa OR "Samoan Islands" OR "Navigator Island" OR "Navigator Islands" OR "Sao Tome" OR "Saudi Arabia" OR Senegal OR Serbia OR Montenegro OR Seychelles OR "Sierra Leone" OR Singapore OR "Sri Lanka" OR Ceylon OR "Solomon Islands" OR Somalia OR "South Africa" OR Sudan OR Suriname OR Surinam OR Swaziland OR Syria OR Tajikistan OR Tadzhikistan OR Tadjikistan OR Tadzhik OR Tanzania OR Thailand OR Togo OR "Togolese Republic" OR Tokelau OR Tonga OR Trinidad OR Tobago OR Tunisia OR Turkey OR Turkmenistan OR Turkmen OR "Turks Caicos" OR "Tuvalu Uganda" OR "United Arab Emirates" OR Uruguay OR Uzbekistan OR Uzbek OR Vanuatu OR "New Hebrides" OR Venezuela OR Vietnam OR "Viet Nam" OR "Virgin Islands" OR "West Bank" OR Yemen OR Yugoslavia OR Zambia OR Zimbabwe))<br>Refined by: Databases: (WOS)<br>**Timespan:** All years.<br>Search language=Auto

Supplemental Table 5

Search terms, notes, and results for Cochrane Library

Boolean operators used between search-term sets	Database searched (date of search)	Fields searched (limitations)
1 AND 2 AND 3 AND 4	Cochrane Library (Jan 21, 2016) http://www.cochranelibrary.com/	Title, abstract, keywords (none)

Results	Search text
71	(“Domestic water” OR “Drinking water” OR “Potable water” OR “Tap water” OR “Water consumption” OR “Well water”) AND (Boil OR Boiled OR Boiling OR Decontamination OR Disinfect OR Disinfectant OR Disinfection OR Domestic OR Household OR Inactivation OR Pasteurise OR Pasteurised OR Pasteurize OR Pasteurized OR “Point of use” OR Point-of-use OR “POU” OR Purification OR Purified OR Purify OR Residential OR “Safe water” OR Treat OR Treated OR Treating OR Treatment OR “Water disinfection” OR “Water purification” OR “Water quality” OR “Water treatment”) AND (“Acute gastrointestinal” OR “AGI” OR “AGII” OR “HCGI” OR “Highly credible gastrointestinal” OR *Campylobacter* OR Cholera OR Cholerae OR Cryptosporidium OR Diarrhea OR Diarrheal OR “Diarrheal disease” OR Diarrheoa OR Diarrheoal OR “Diarrheoal disease” OR Dysentery OR “E. coli” OR Enteric OR “Enteric virus” OR “Enteric viruses” OR Enterovirus OR Enteroviruses OR “E. coli” OR “Escherichia coli” OR Gastrointestinal OR “Gastro intestinal” OR Gastro-enteric OR *Giardia* OR Helicobacter OR Helminth OR Hepatitis OR Intestinal OR Norovirus OR “Norwold-like virus” OR Rotavirus OR *Salmonella* OR Shigella OR “Vibrio cholera” OR “Vibrio cholerae” OR Waterborne OR Water-borne OR “Waterborne pathogen” OR “Waterborne pathogens”) AND (Afghanistan OR Algeria OR Angola OR Anguilla OR Antigua OR Barbuda OR Argentina OR Armenia OR Armenian OR Aruba OR Azerbaijan OR Bahamas OR Bahrain OR Bangladesh OR Barbados OR Benin OR Byelarus OR Byelorussian OR Belarus OR Belorussian OR Belorussia OR Belize OR Bhutan OR Bolivia OR Botswana OR Brazil OR Brunei OR Burkina Faso OR “Burkina Fasso” OR “Upper Volta” OR Burundi OR Urundi OR Cambodia OR “Khmer Republic” OR Kampuchea OR Cameroon OR Cameroons OR Cameron OR Camerons OR “Cape Verde” OR “Cayman Islands” OR “Central African Republic” OR Chad OR Chile OR China OR Colombia OR Comoros OR “Comoro Islands” OR Comores OR Mayotte OR Congo OR Zaire OR “Cook Islands” OR “Costa Rica” OR “Cote dIvoire” OR “Ivory Coast” OR Croatia OR Cuba OR Cyprus OR Djibouti OR “French Somaliland” OR Dominica OR “Dominican Republic” OR “East Timor” OR “East Timur” OR “Timor Leste” OR Ecuador OR Egypt OR “United Arab Republic” OR “El Salvador” OR Eritrea OR Ethiopia OR “Falkland Islands” OR “Las Malvinas” OR Fiji OR Gabon OR “Gabonese Republic” OR Gambia OR Gaza OR “Georgia Republic” OR “Georgian Republic” OR Ghana OR “Gold Coast” OR Greece OR Grenada OR Guatemala OR Guinea OR Guam OR Guadeloupe OR Guiana OR Guyana OR Haiti OR Honduras OR “Hong Kong” OR India OR Maldives OR Indonesia OR Iran OR Iraq OR Jamaica OR Jordan OR Kazakhstan OR Kazakh OR Kenya OR Kiribati OR Korea OR Kosovo OR Kuwait OR Kyrgyzstan OR Kirghizia OR “Kyrgyz Republic” OR Kirghiz OR Kirgizstan OR “Lao PDR” OR Laos OR Lebanon OR Lesotho OR Basutoland OR Liberia OR Libya OR Macau OR Madagascar OR “Malagasy Republic” OR Maldives OR Malaysia OR Malaya OR Malay OR Sabah OR Sarawak OR Malawi OR Nyasaland OR Mali OR Malta OR “Marshall Islands” OR Martinique OR Mauritania OR Mauritius OR “Agalega Islands” OR Mexico OR Micronesia OR “Middle East” OR Mongolia OR Montserrat OR Morocco OR Ifni OR Mozambique OR Myanmar OR Myanma OR Burma OR Namibia OR Nauru OR Nepal OR Niui OR “Netherlands Antilles” OR “New Caledonia” OR Nicaragua OR Niger OR Nigeria OR “Northern Mariana Islands” OR Oman OR Mayotte OR Muscat OR Pakistan OR Palau OR Palestine OR Panama OR Paraguay OR Peru OR Philippines OR Philipines OR Phillipines OR Phillippines OR Polynesia OR “Puerto Rico” OR Qatar OR Reunion OR Rwanda OR Ruanda OR “Saint Kitts” OR “St Kitts” OR Nevis OR “Saint Lucia” OR “St Lucia” OR “Saint Vincent” OR “St Vincent” OR Grenadines OR Samoa OR “Samoan Islands” OR “Navigator Island” OR “Navigator Islands” OR “Sao Tome” OR “Saudi Arabia” OR Senegal OR Serbia OR Montenegro OR Seychelles OR “Sierra Leone” OR Singapore OR “Sri Lanka” OR Ceylon OR “Solomon Islands” OR Somalia OR “South Africa” OR Sudan OR Suriname OR Surinam OR Swaziland OR Syria OR Tajikistan OR Tadzhikistan OR Tadjikistan OR Tadzhik OR Tanzania OR Thailand OR Togo OR “Togolese Republic” OR Tokelau OR Tonga OR Trinidad OR Tobago OR Tunisia OR Turkey OR Turkmenistan OR Turkmen OR “Turks Caicos” OR “Tuvalu Uganda” OR “United Arab Emirates” OR Uruguay OR Uzbekistan OR Uzbek OR Vanuatu OR “New Hebrides” OR Venezuela OR Vietnam OR “Viet Nam” OR “Virgin Islands” OR “West Bank” OR Yemen OR Yugoslavia OR Zambia OR Zimbabwe)

Supplemental Table 6

Criteria and associated scoring used to assess likely impact of bias and/or poor study quality

Criteria	Score = 0 (bias or poor quality likely)	Score = 1 (uncertain about bias/quality)	Score = 2 (bias or poor quality less likely
1. Country and region where study conducted	Neither specified	Country but not region specified	Both country and region specified
2. Study year and data collection period reported	Neither year nor duration specified	Year or duration specified	Both year and duration specified
3. Random sampling/selection used	No or not specified	Possibly, but unclear	Yes, clearly stated
4. Sampling/selection method clearly described	No	Yes, but unclear	Yes
5.1. Health outcome assessment protocol described	No	Yes	
5.2. Health outcome assessed directly or reported	Reported (by or for participant)	Direct measurement (blood, stool, other samples, or tests)	
6.1. 2 × 2 table (or data needed to construct one) provided	No	Yes	
6.2. Adjusted effect estimates provided	No	Yes	

Supplemental Table 7

Completed PRISMA 2009 checklist

Section/topic	#	Checklist item	Reported on page #
**TITLE**			
Title	1	Identify the report as a systematic review, meta-analysis, or both.	Title page
**ABSTRACT**			
Structured summary	2	Provide a structured summary including, as applicable: background; objectives; data sources; study eligibility criteria, participants, and interventions; study appraisal and synthesis methods; results; limitations; conclusions and implications of key findings; systematic review registration number.	1
**INTRODUCTION**			
Rationale	3	Describe the rationale for the review in the context of what is already known.	2–3
Objectives	4	Provide an explicit statement of questions being addressed with reference to participants, interventions, comparisons, outcomes, and study design (PICOS).	4
**METHODS**			
Protocol and registration	5	Indicate if a review protocol exists, if and where it can be accessed (e.g., Web address), and, if available, provide registration information including registration number.	–
Eligibility criteria	6	Specify study characteristics (e.g., PICOS, length of follow-up) and report characteristics (e.g., years considered, language, publication status) used as criteria for eligibility, giving rationale.	4
Information sources	7	Describe all information sources (e.g., databases with dates of coverage, contact with study authors to identify additional studies) in the search and date last searched.	4
Search	8	Present full electronic search strategy for at least one database, including any limits used, such that it could be repeated.	Supplemental Tables 2–5
Study selection	9	State the process for selecting studies (i.e., screening, eligibility, included in systematic review, and, if applicable, included in the meta-analysis).	4–5

(continued)

Supplemental Table 7
Continued

Section/topic	#	Checklist item	Reported on page #
Data collection process	10	Describe method of data extraction from reports (e.g., piloted forms, independently, in duplicate) and any processes for obtaining and confirming data from investigators.	5–6
Data items	11	List and define all variables for which data were sought (e.g., PICOS, funding sources) and any assumptions and simplifications made.	4–5
Risk of bias in individual studies	12	Describe methods used for assessing risk of bias of individual studies (including specification of whether this was done at the study or outcome level), and how this information is to be used in any data synthesis.	6–7
Summary measures	13	State the principal summary measures (e.g., risk ratio, difference in means).	5
Synthesis of results	14	Describe the methods of handling data and combining results of studies, if done, including measures of consistency (e.g., I^2) for each meta-analysis.	5–7
Risk of bias across studies	15	Specify any assessment of risk of bias that may affect the cumulative evidence (e.g., publication bias, selective reporting within studies).	6–7
Additional analyses	16	Describe methods of additional analyses (e.g., sensitivity or subgroup analyses, meta-regression), if done, indicating which were pre-specified.	6–7
**RESULTS**			
Study selection	17	Give numbers of studies screened, assessed for eligibility, and included in the review, with reasons for exclusions at each stage, ideally with a flow diagram.	8–10
Study characteristics	18	For each study, present characteristics for which data were extracted (e.g., study size, PICOS, follow-up period) and provide the citations.	8–13
Risk of bias within studies	19	Present data on risk of bias of each study and, if available, any outcome level assessment (see item 12).	11–13
Results of individual studies	20	For all outcomes considered (benefits or harms), present, for each study: (a) simple summary data for each intervention group (b) effect estimates and confidence intervals, ideally with a forest plot.	14–21
Synthesis of results	21	Present results of each meta-analysis done, including confidence intervals and measures of consistency.	14–21
Risk of bias across studies	22	Present results of any assessment of risk of bias across studies (see Item 15).	21 and Supplemental Tables 8–10
Additional analysis	23	Give results of additional analyses, if done (e.g., sensitivity or subgroup analyses, meta-regression [see Item 16]).	Supplemental Tables 6–7
**DISCUSSION**			
Summary of evidence	24	Summarize the main findings including the strength of evidence for each main outcome; consider their relevance to key groups (e.g., healthcare providers, users, and policy makers).	22–28
Limitations	25	Discuss limitations at study and outcome level (e.g., risk of bias), and at review-level (e.g., incomplete retrieval of identified research, reporting bias).	26–27

(continued)

Supplemental Table 7

Continued

Section/topic	#	Checklist item	Reported on page #
Conclusions	26	Provide a general interpretation of the results in the context of other evidence, and implications for future research.	27–28
**FUNDING**			
Funding	27	Describe sources of funding for the systematic review and other support (e.g., supply of data); role of funders for the systematic review.	29

Source: **http://www.prisma-statement.org/documents/PRISMA%202009%20checklist.doc**

From: Moher D, Liberati A, Tetzlaff J, Altman DG, The PRISMA Group (2009). Preferred Reporting Items for Systematic Reviews and Meta-Analyses: The PRISMA Statement. PLoS Med 6(7): e1000097. doi:10.1371/journal.pmed1000097

Page numbers reference the submitted manuscript and may not align with the published version.

Supplemental Table 8

Meta-regression results for studies reporting protozoal and diarrheal outcomes

	Protozoal outcome studies (*N* = 11)				Diarrheal outcome studies (*N* = 7)			
Variables	OR	95% CI	*P* value	Monte Carlo *P* value	OR	95% CI	*P* value	Monte Carlo *P* value
Total participants (or households)	1.00	0.99–1.01	0.054	0.02	1.00	0.99 to 1.00	0.69	0.65
Participant age < 15 (vs. adults)	1.16	0.36–3.74	0.74	0.73	0.19	0.00 to > 3k	0.28	0.19
U participants (vs. only R)	1.40	0.22–9.02	0.65	0.60	--	--	--	--
U and R participants (vs. only R)	1.46	0.18–12.05	0.64	0.61	--	--	--	--
Outbreak investigation (vs. other)	-	-	-	-	--	--	--	--
Study duration (months)	0.98	0.94–1.01	0.17	0.10	0.98	0.44to 2.20	0.79	0.44
Random sampling (vs. no)	0.12	0.01–2.49	0.12	0.08	1.47	0.01 to 332	0.53	0.56
Outcome measured (vs. reported)	-	-	-	-	0.89	0.00 to 713	0.86	0.89

CI = confidence interval; OR = odds ratio; R = rural; U = urban; k = 1,000. Cells with "-" indicate instances where there was too much collinearity with the associated covariate. Cells with "--" indicate instances where there were an insufficient number of observations available.

Supplemental Table 9

Meta-regression results for studies reporting protozoal and diarrheal outcomes: controlling for study quality

	Protozoal outcome studies (*N* = 11)				Diarrheal outcome studies (*N* = 7)			
Variables	OR	95% CI	*P* value	Monte Carlo *P* value	OR	95% CI	*P* value	Monte Carlo *P* value
Total participants (or households)	1.00	0.99–1.00	0.42	0.43	1.00	0.99 to 1.00	0.53	0.48
Participant age < 15 (vs. adults)	1.24	0.16–9.68	0.79	0.72	0.21	0.00 to 665.7	0.25	0.09
U participants (vs. only R)	4.56	0.35–59.5	0.18	0.14	--	--	--	--
U and R participants (vs. only R)	1.33	0.01–127.7	0.87	0.93	--	--	--	--
Outbreak investigation (vs. other)	-	-	-	-	6.82	0.00 to > 999k	0.59	0.67
Study duration (months)	0.97	0.86–1.08	0.47	0.42	0.76	0.00 to 88.9	0.60	0.59
Quality index grade high (vs. medium/low)	3.53	0.03–480.3	0.52	0.47	0.72	0.00 to > 200k	0.79	0.72

CI = confidence interval; OR = odds ratio; R = rural; U = urban; k = 1,000. Cells with "-" indicate instances where there was too much collinearity with the associated covariate. Cells with "--" indicate instances where there were an insufficient number of observations available

Supplemental Table 10

Assessment of likely impact of bias and/or poor study quality: Results for each study

		Bias/quality criteria									
						5		6			
Author	Year	1	2	3	4	5.1	5.2	6.1	62	Quality index*	Grade†
Aggarwal	2002	2	2	2	2	1	1	0	0	8.3	High
Al-Delaimy	2014	2	2	0	2	1	1	1	0	7.5	High
Bello	2011	2	2	0	2	1	1	0	1	7.5	High
Cardenas	1993	2	2	2	0	1	0	1	0	6.7	Medium
Carrero	2013	2	1	0	2	1	1	1	0	6.7	Medium
Choy	2014	2	2	2	2	1	1	0	1	9.2	High
Cifuentes	1998	2	2	1	0	1	1	1	0	6.7	Medium
Cohen	2015	2	2	2	2	1	0	0	0	7.5	High
Corwin	1995	2	2	1	0	1	1	1	0	6.7	Medium
Fredrick	2015	2	2	1	2	1	1	1	1	9.2	High
Gunawardena	2004	2	2	2	0	1	1	0	1	7.5	High
Herrera	2006	2	2	1	1	1	1	1	0	7.5	High
Iijima	2001	2	2	1	1	1	0	1	0	6.7	Medium
Kelly	1997	2	2	1	0	1	1	0	0	5.8	Low
Knight	1992	2	2	2	2	1	1	0	1	9.2	High
Lee	2012	2	2	1	1	1	1	1	0	7.5	High
Li	2007	2	1	2	0	1	1	0	1	6.7	Medium
Marcano	2013	2	1	1	0	1	1	1	0	5.8	Low
Nunez	2003	2	1	1	0	1	1	1	0	5.8	Low
Psutka	2013	2	1	2	2	0	0	0	0	5.8	Low
Ries	1992	2	2	1	2	1	1	1	1	9.2	High
Rondon	2003	2	2	1	1	1	1	1	0	7.5	High
Sarkar	2014a	2	2	1	1	1	1	0	1	7.5	High
Sarkar	2014b	2	2	1	0	1	1	0	1	6.7	Medium
Sharma	2009	2	2	2	1	1	1	1	1	9.2	High
Weber	1994	2	2	1	0	1	1	1	0	6.7	Medium
Wordemann	2006	2	2	2	1	1	1	0	0	7.5	High

* Quality index calculating as a composite index of criteria 1–6, using equal weights and then converted to a 0–10 scale.
† Low score: < 6; medium score: 6–7; high score: ≥ 7.

Supplemental Table 11

Pooled OR by outcome and stratified by estimated study quality grade

	Low		Medium		High	
Study quality grade	Pooled OR (*n*)	95% CI	Pooled OR (*n*)	95% CI	Pooled OR (*n*)	95% CI
Bacterial						
Vibrio cholerae			0.53 (2)	0.29–0.96	0.11 (2)	0.01–1.12
Helicobacter pylori					1.12 (1)	0.58–2.16
Salmonella typhi					1.30 (1)	0.62–2.71
Helminthic						
Multiple helminthic infections					1.21 (2)	0.80–1.81
Ascaris					1.18 (2)	0.09–14.9
Strongyloides stercoralis					0.30 (1)	0.12–0.76
Trichuris					1.62 (1)	0.46–5.70
Hookworm					1.16 (1)	0.26–5.10
Protozoal						
Giardia	0.27 (1)	0.12–0.59			0.84 (3)	0.43–1.62
Blastocystis			0.17 (2)	0.03–0.94	0.40 (1)	0.19–0.86
Multiple protozoan parasites	0.80 (1)	0.45–1.43			0.83 (1)	0.32–2.16
Cryptosporidium parvum					0.49 (1)	0.25–0.97
Entamoeba histolytica/dispar					1.49 (1)	0.19–11.6
Viral						
Hepatitis E			0.77 (1)	0.51–1.17	1.11 (1)	0.61–1.97
Rotavirus			0.83 (2)	0.65–1.05		
Diarrheal						
Diarrheal disease outcomes	0.60 (2)	0.40–0.89	0.60 (3)	0.50–0.78	0.31 (2)	0.05–2.03

CI = confidence interval; OR = odds ratio.
